# Formulation Optimization and Comprehensive Performance Evaluation of Waterborne Acrylic Road Marking Paints via Orthogonal Experiment and Weighted Comprehensive Scoring

**DOI:** 10.3390/polym18161935

**Published:** 2026-08-07

**Authors:** Zhi Zheng, Naisheng Guo, Hongbin Zhu, Xiaoqing Wang, Haoliang Li, Jincheng Wang, Zidong Zhou, Xuelian Li

**Affiliations:** 1 College of Transportation Engineering, Dalian Maritime University, Dalian 116026, China; zhizheng@dlmu.edu.cn (Z.Z.); zhuhongbin@dlmu.edu.cn (H.Z.); lihaoliang@dlmu.edu.cn (H.L.); 2 College of Architecture and Civil Engineering, Xi’an University of Science and Technology, Xi’an 710054, China; wangxiaoqing@xust.edu.cn; 3 Beijing Key Laboratory of Traffic Engineering, Beijing University of Technology, Beijing 100124, China; wjc2131243749@163.com; 4 School of Traffic and Transportation Engineering, Changsha University of Science and Technology, Changsha 410114, China; zhouzidong@stu.csust.edu.cn

**Keywords:** waterborne acrylic road marking paint, hot-melt road marking paint, road traffic marking, orthogonal experiment, weighted comprehensive scoring, retroreflected luminance, abrasion resistance, durability, VOC emissions

## Abstract

Conventional solvent-based and hot-melt road marking paints face significant challenges regarding high volatile organic compound (VOC) emissions and limited durability, necessitating the development of eco-friendly, high-performance alternatives. In this study, a waterborne acrylic road marking paint was systematically formulated and optimized using an L_16_(4^5^) orthogonal experimental design coupled with a comprehensive weighted scoring method integrating subjective and objective (entropy) weights. Four key formulation parameters (pigment-to-binder ratio, titanium dioxide content, ground calcium carbonate content, and coalescing agent dosage) were investigated, with abrasion resistance, hiding power, luminance factor, and stain resistance as evaluation criteria. The optimized formulation was identified through range analysis of comprehensive scores and subsequently subjected to rigorous performance characterization, including retroreflectivity optimization, Taber and accelerated abrasion testing, UV-accelerated weathering, skid resistance, and VOC emissions measurement using a self-designed sealed chamber system. Benchmark comparisons against commercial waterborne and hot-melt paints demonstrated that the developed formulation achieves superior abrasion resistance, exceptional weatherability, and meaningfully lower VOC emissions. Field application on an operational highway section in Liaoning Province, China, confirmed the practical constructability and performance reliability of the optimized paint under real-world construction conditions. This research provides both theoretical guidance and practical validation for the design of sustainable, durable, and highly visible road marking materials, contributing to the advancement of environmentally responsible transportation infrastructure.

## 1. Introduction

Road traffic markings play a critical role in traffic safety by providing continuous visual guidance to drivers, especially under nighttime or adverse weather conditions [1,2]. The visibility, durability, and skid resistance of marking paints directly influence accident rates and overall traffic efficiency [3,4,5]. Traditional road marking paints, such as solvent-based and hot-melt types, have been widely used due to their good retroreflectivity and mechanical strength [6,7]. However, solvent-based paints release large amounts of volatile organic compounds (VOCs) during application, posing serious health and environmental risks [8]. Hot-melt paints, although solvent-free, require high-temperature heating (160–250 °C) during construction, leading to significant energy consumption and potential thermal degradation of organic components, which in turn generates hazardous emissions [9,10]. Furthermore, traditional hot-melt and solvent-based coatings often suffer from accelerated degradation, premature cracking, and peeling under continuous environmental weathering and traffic loads, which severely compromise their long-term retroreflectivity and structural integrity [11,12]. In response to stringent environmental regulations and the global pursuit of low-carbon infrastructure, waterborne road marking paints have emerged as a promising alternative, offering low VOC emissions, ease of application, and good adhesion to various pavement surfaces [13,14].

Despite their environmental advantages, waterborne acrylic road marking paints often face performance challenges that limit their widespread adoption, particularly under high-demand traffic conditions [15]. Key limitations include relatively low abrasion resistance, insufficient retroreflectivity retention, poor stain resistance, and inadequate weatherability compared to their solvent-based or hot-melt counterparts [16,17,18]. These shortcomings primarily stem from the intrinsic properties of waterborne systems, namely water evaporation kinetics, coalescence of polymer particles, and the interaction between the organic binder and inorganic fillers, all of which differ fundamentally from solvent-borne and hot-melt systems. The pigment-to-binder (P/B) ratio, filler types and contents, and coalescing agent dosage are known to critically influence the film formation process, mechanical integrity, and optical performance of waterborne coatings [14,19]. Most existing studies varied one or two formulation parameters at a time, without considering interactive effects among multiple components [10]. As a result, the derived “optimum” formulations are often locally optimal rather than globally optimal. Moreover, performance evaluation of road marking paints typically involves several indicators (e.g., abrasion resistance, hiding power, luminance factor, stain resistance, retroreflectivity, skid resistance, and VOC emissions). Optimizing multiple formulation parameters simultaneously to achieve a balanced trade-off among conflicting performance indicators (e.g., high hiding power vs. high abrasion resistance, or good retroreflectivity vs. low VOC emissions) remains a nontrivial task [20]. Few studies have employed a comprehensive scoring method to integrate these diverse, often conflicting, indicators into a single objective function, making it difficult to compare formulations holistically.

Previous studies have explored various aspects of waterborne paints. Several researchers investigated the effects of different resin systems, such as styrene-acrylic and pure acrylic emulsions, on adhesion and weather resistance [21,22]. Titanium dioxide (TiO_2_), owing to its high refractive index, has been widely adopted in waterborne coatings, and substituting a portion of TiO_2_ with calcined kaolin, calcite, or other industrial mineral fillers has been investigated as a cost-reduction strategy [23]. However, such substitution can adversely affect the chromaticity, hiding power, and mechanical properties of the coatings. More recently, the amount and ratio of flake waste glass powder and rutile TiO_2_ were designed based on the pigment volume concentration (PVC) and critical pigment volume concentration (CPVC) theory, and it was found that when the filler amount was 20 wt% and the ratio was 2:1 (PVC: CPVC), the tensile strength of the coatings increased by 49% and water absorption reduced by 15% [24]. The effect of different pigment/extenders (including calcium carbonate, dolomite, and kaolin) on the stability of high-PVC water-based paints has also been thoroughly studied, revealing that paint dispersions based on carbonate extenders show syneresis and soft settling, which is easily mixable [25,26]. The influence of coalescing agents on the minimum film-forming temperature (MFFT) and mechanical properties of waterborne coatings has also been well documented [27]. Moreover, glass beads as retroreflective elements have been extensively studied, with findings indicating that bead size, refractive index, and embedment depth significantly affect the coefficient of retroreflected luminance [28,29]. In terms of durability, accelerated abrasion tests (i.e., Taber abrasion method) and UV aging protocols have been employed to evaluate wear resistance and weather resistance, respectively [12,30,31]. While many laboratory-scale optimizations have been reported, real-world application data, especially under actual traffic and environmental conditions, are scarce. The transferability of laboratory results to field performance remains questionable.

Despite these research efforts, several critical knowledge gaps remain unaddressed. First, most existing studies have varied formulation parameters in a one-factor-at-a-time manner, without systematically considering the interactive effects among multiple components such as the pigment-to-binder ratio, TiO_2_ content, filler dosage, and coalescing agent concentration. As a result, the derived “optimum” formulations are often locally rather than globally optimal. Second, while individual performance indicators such as abrasion resistance or hiding power have been extensively studied, a holistic evaluation framework that integrates multiple, often conflicting, performance criteria (mechanical durability, optical performance, weatherability, and environmental impact) into a unified optimization objective remains lacking. Third, although accelerated laboratory tests are commonly employed to assess paint durability, systematic comparisons with commercial benchmark products under identical conditions, coupled with real-world field validation, are scarce. The transferability of laboratory-optimized formulations to actual engineering practice remains questionable.

To address these gaps, this study presents a systematic formulation optimization and comprehensive performance evaluation of waterborne acrylic road marking paints. Specifically, an L_16_(4^5^) orthogonal experimental design was employed to investigate four key formulation factors: P/B ratio, TiO_2_ content, ground calcium carbonate (GCC) content, and coalescing agent dosage. Four performance indicators (abrasion resistance, hiding power, luminance factor, and stain resistance) were selected as evaluation criteria. To combine these indicators into a single comprehensive score, a hybrid weighting method was established by integrating subjective engineering judgment with objective entropy weights. The optimal formulation was then identified through range analysis of the comprehensive scores. Subsequently, the optimized waterborne paint was subjected to a series of comprehensive laboratory tests, including retroreflectivity at different glass bead contents, Taber abrasion, self-developed accelerated abrasion, UV-accelerated weathering, wet-skid resistance, and VOC emissions using a self-designed sealed chamber system. For comparison, commercial waterborne and hot-melt road marking paints were tested under identical conditions. Finally, field application was carried out on an actual highway section in Liaoning Province, China, to validate the constructability and performance reliability of the developed paint under real-world conditions.

## 2. Materials and Methods

### 2.1. Materials

The film-forming material (waterborne acrylic resin emulsion), wetting and dispersing agent (acrylic copolymer sodium salt), and defoamer (silicone-based) were all supplied by Shanghai Yike New Material Technology Co., Ltd. (Shanghai, China). The main technical properties of the Type 538 waterborne acrylic resin emulsion used in this study are summarized in Table 1. This emulsion exhibits strong photochemical and chemical stability, along with outstanding weatherability, cracking resistance, stain resistance, and fast-drying performance. Its relatively high solids content (48~50%) and low MFFT (25 °C) enable rapid film formation at ambient temperatures, which is critical for road marking applications where quick drying to traffic is required. The wetting and dispersing agent used in this study was an acrylic copolymer sodium salt, as recommended by the supplier for use with the Type 538 waterborne acrylic resin emulsion and the specific pigment/filler combination. This dispersant was selected after preliminary screening tests confirmed its adequate dispersion efficiency (Hegman fineness ≤ 15 μm), good storage stability (no significant settling after 7 days), and low foaming tendency during high-speed dispersion. It should be noted, however, that for waterborne road marking paints, dispersants with ammonium counterions are generally preferred over sodium salts. During film drying, the ammonium ion volatilizes as ammonia, leaving behind an acid-functional polymer that is less water-sensitive and more hydrophobic. In contrast, sodium counterions remain in the dried film as non-volatile hydrophilic species, which can increase the water sensitivity of the cured film and potentially retard drying. The sodium salt dispersant was selected in this study primarily for practical reasons—supplier recommendation for the specific resin system, preliminary screening results showing comparable dispersion quality, and availability/cost considerations at the time of formulation development. We acknowledge that replacing the sodium salt dispersant with an ammonium salt counterpart represents a promising direction for future formulation refinement to further enhance drying speed and water resistance.

**Figure 1:**
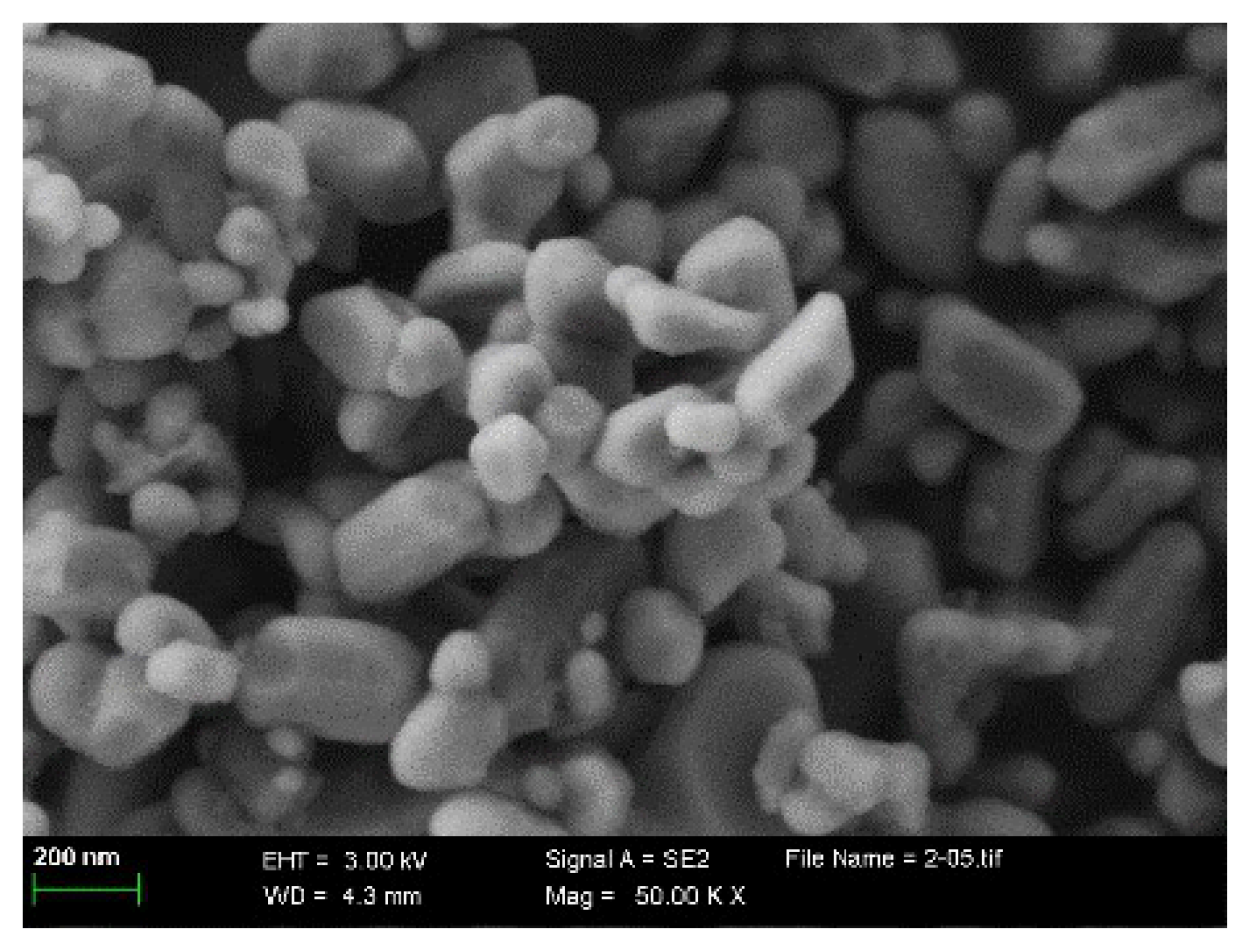
Macro and micro morphology of the R-996 TiO_2_ powder.

Rutile TiO_2_ was selected as the white pigment due to its high refractive index (2.78), excellent opacity, and good weather resistance. The well-dispersed TiO_2_ particles, owing to their high intrinsic hardness (Mohs ~6–6.5), act as rigid micro-reinforcements within the polymer matrix, deflecting and dissipating abrasive contact energy. The key technical properties of the TiO_2_ pigment used in this study are summarized in Table 2. The pigment exhibits a mean particle size (D_50_) of approximately 0.27 μm with a relatively narrow size distribution (D_10_ ≈ 0.15 μm, D_90_ ≈ 0.45 μm), which is favorable for achieving high light scattering efficiency and good dispersion stability. The pigment particles are surface-coated with inorganic oxides (Al_2_O_3_ at 3~5 wt% and SiO_2_ at 2~3 wt%) combined with an organic surface treatment to enhance dispersibility in the acrylic emulsion system and to improve weatherability by reducing photocatalytic activity. The BET specific surface area of the pigment is 12~15 m^2^/g, consistent with values reported for high-performance rutile TiO_2_ grades. Scanning electron microscopy (SEM) was employed to characterize the morphology of the TiO_2_ pigment, and the results are presented in Figure 1. The SEM micrographs reveal that the pigment particles exhibit a rounded ellipsoidal morphology with relatively uniform size distribution, which is conducive to effective wetting and dispersion within the waterborne acrylic resin emulsion.

**Figure 2:**
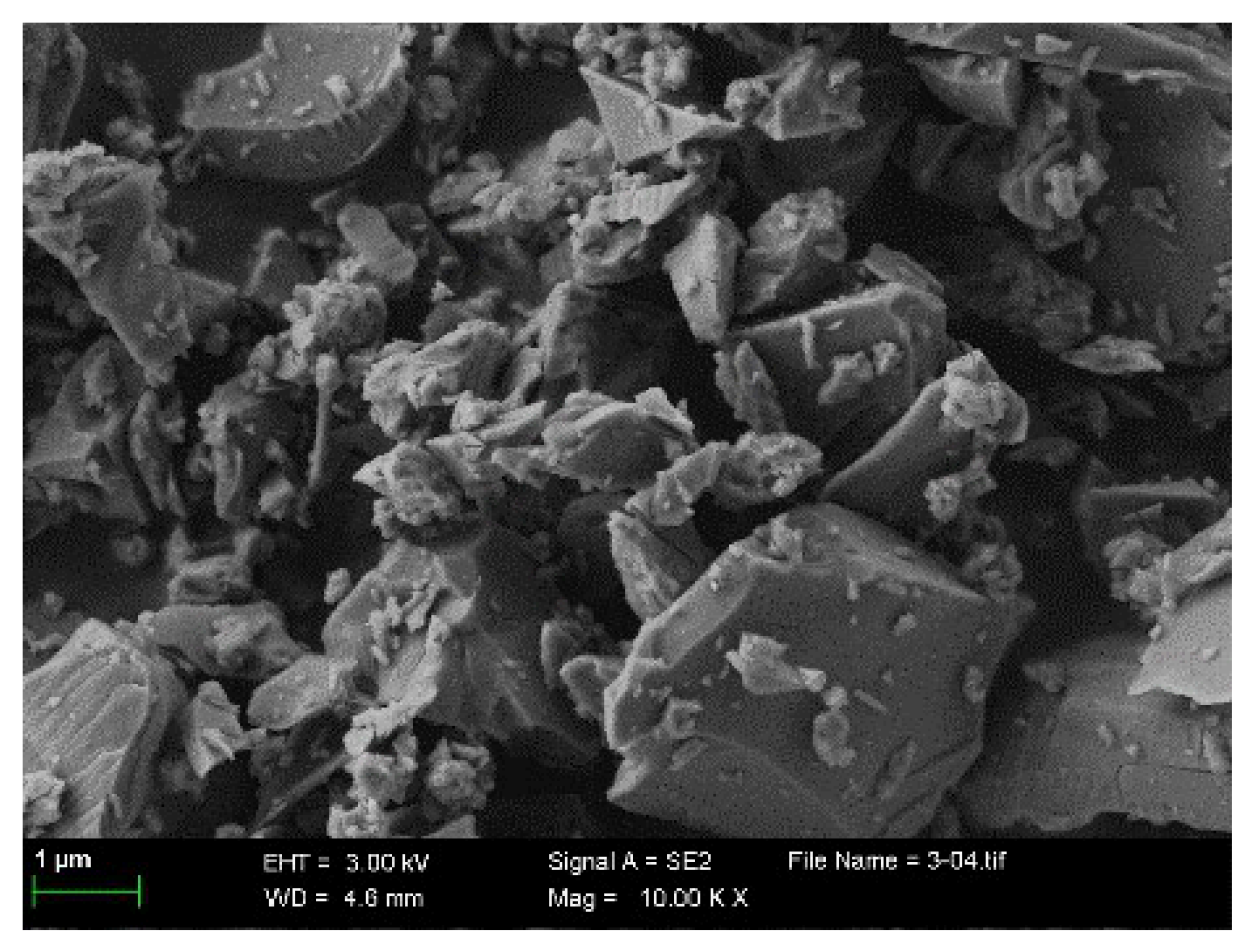
Macro and micro morphology of the GCC powder.

GCC was utilized as the extender filler. Its primary technical properties are summarized in Table 3. The GCC exhibits a mean particle size (D_50_) of approximately 4.5 μm with a particle size distribution of D_10_ ≈ 1.5 μm and D_90_ ≈ 18 μm (by laser diffraction), and an oil absorption value of 18~20 g/100 g (ASTM D281 [32]). These parameters correspond to a GCC grade with a maximum particle size of approximately 18 μm (D_90_), which is the grade commonly employed in road marking paint formulations. SEM was employed to observe the morphology of the GCC, with the results presented in Figure 2. As illustrated in Figure 2, the particles display a flake-like (plate-like) morphology, which is advantageous for improving the opacity, hardness, and wear resistance of the coating. Specifically, GCC is a relatively soft extender (Mohs ~3) that does not directly enhance abrasion resistance through hardness; however, at optimal loading levels, it can improve film densification by occupying interstitial voids, thereby indirectly contributing to mechanical integrity.

**Table 2:** Main technical parameters of the TiO_2_ powder.

Items	Standard	Results
Density/(g·cm^−3^)	ISO787-10 [33]	4.0
TiO_2_ content/%	ISO 591-1 [34]	≥93
Mean particle size (D_50_)/μm	Laser diffraction	~0.27
Particle size distribution/μm	Laser diffraction	D_10_ ≈ 0.15, D_90_ ≈ 0.45
BET specific surface area/(m^2^·g^−1^)	ISO 9277 [35]	12~15
Surface coating	-	Al_2_O_3_ (3~5 wt%) + SiO_2_ (2~3 wt%) + organic treatment
Volatiles at 105 °C/%	ISO787-2 [36]	≤0.5
Water-soluble matter/%	ISO 787-2 [36]	≤0.5
Residue on 45 μm sieve/%	ISO 787-18 [37]	≤0.1
Oil absorption value/(g·100 g^−1^)	ISO 787-5 [38]	≤22
pH of aqueous suspension	ISO 787-9 [39]	6.5–8.5
Resistivity of aqueous extract/(Ω·m)	ISO 787-14 [40]	≥100

**Table 3:** Key properties of the GCC powder.

Appearance	Whiteness /%	Density /(g·cm^−3^)	D_50_/μm	Particle Size Distribution/μm	Oil Absorption/(g·100 g^−1^)	Active Ingredient Content/%
White powder	95	2.92	~ 4.5	D_10_ ≈ 1.5, D_90_ ≈ 18	18~20	98.5

Propylene glycol phenyl ether (PPH) was selected as the coalescing agent in this study. PPH is a high-boiling-point (242.7 °C), low-water-solubility glycol ether with strong solvency for acrylic, styrene-acrylic, and vinyl acetate polymers. Compared to the industry-standard coalescing agent 2,2,4-trimethyl-1,3-pentanediol monoisobutyrate (commonly known as alcohol ester-12), PPH offers several advantages for waterborne acrylic road marking paint formulations. First, PPH achieves comparable or superior film-forming performance at significantly lower dosages—approximately 30–50% reduction in dosage alcohol ester-12 under equivalent film quality conditions (same gloss, leveling, sag resistance, color development, and scrub resistance). The comprehensive film-forming efficiency of PPH is reportedly 1.5~2 times that of conventional coalescents, resulting in substantial production cost savings. Second, PPH exhibits excellent compatibility with acrylic-based latexes, which is the binder system employed in this study. Third, PPH has a low vapor pressure and low volatility, contributing to reduced VOC emissions from the formulated coating, and is considered non-toxic and environmentally friendly, aligning with the environmental objectives of this work. The effectiveness of PPH in waterborne road marking paint formulations has also been demonstrated in previous studies. Based on these considerations, PPH was chosen as the preferred coalescing agent for the acrylic emulsion system used in this study. The solvents used in this study included absolute ethanol and aqueous ammonia (analytical grade), along with other analytical grade reagents. Deionized water was prepared in the laboratory.

According to the Chinese National Standard GB/T 24722-2020 [41], glass beads used for road markings are categorized into four grades (Types 1 to 4) depending on their particle size distribution, where larger type numbers indicate larger particle sizes. The standard recommends Type 1 glass beads as drop-on materials for hot-melt, two-component, and waterborne road marking paints. Therefore, Type 1 glass beads were chosen as the drop-on reflective material for this research. As shown in Table 4, laboratory testing confirmed that the performance properties of the selected Type 1 glass beads meet all the specified technical requirements of GB/T 24722-2020.

### 2.2. Methods

#### 2.2.1. Paint Preparation

All specimens were prepared and cured in a temperature- and humidity-controlled laboratory maintained at 23 ± 2 °C and 50 ± 5% relative humidity. These conditions were maintained throughout the entire preparation process to ensure reproducibility and to minimize the influence of environmental fluctuations on dispersion quality and emulsion stability. First, the waterborne acrylic resin emulsion was placed into a disperser vessel and stirred at 500 rpm. A partial amount of the predetermined aqueous ammonia was added to adjust the system’s pH, followed by the sequential addition of wetting and dispersing agents and defoamers. To avoid the agglomeration of pigments and fillers, they were gradually added in batches under continuous agitation, ensuring no noticeable lumps formed during each addition. Upon completion of the solid addition, the stirring speed was elevated to 1000–1500 rpm to disperse the mixture at high speed for 20 min under the aforementioned controlled environmental conditions. The speed was then reduced to 800 rpm to slowly incorporate deionized water and other liquid components (e.g., coalescing agents). Once all ingredients were completely added, the speed was restored to 1000 rpm for a further 20 min of stirring. Finally, after in-process quality control confirmed that all parameters were acceptable, the prepared paint was filtered through a sieve with an aperture of approximately 125 μm and packaged.

#### 2.2.2. Abrasion Resistance Test

(1) Taber Abrasion Test

Abrasion resistance was evaluated according to the Chinese National Standard GB/T 1768-2006 [42]. The coating was applied to tinplate panels (approximately 100 mm in diameter, featuring a 6.35 mm central hole) using a drawdown bar to achieve a dry film thickness of 25–30 μm. It should be noted that the Taber abrasion test was performed on bare coating specimens without drop-on glass beads, as the standard method is designed to evaluate the intrinsic abrasion resistance of the coating film itself. A coated specimen was fixed onto a rotating turntable. Two abrasive rubber wheels, each bearing a 500 g load, applied constant pressure onto the surface of the specimen. During rotation at 60 ± 2 rpm, the wheels created an annular wear track, determining the wear resistance of the material via continuous frictional contact. The abraded surface area is approximately 30 cm^2^ per specimen, as determined by the geometry of the abrasive wheels and the turntable configuration specified in GB/T 1768-2006 [42]. The abrasion resistance of the coating was expressed as the mass loss of the paint film following a predetermined number of abrasion cycles.

(2) Accelerated Abrasion Test

**Figure 3:**
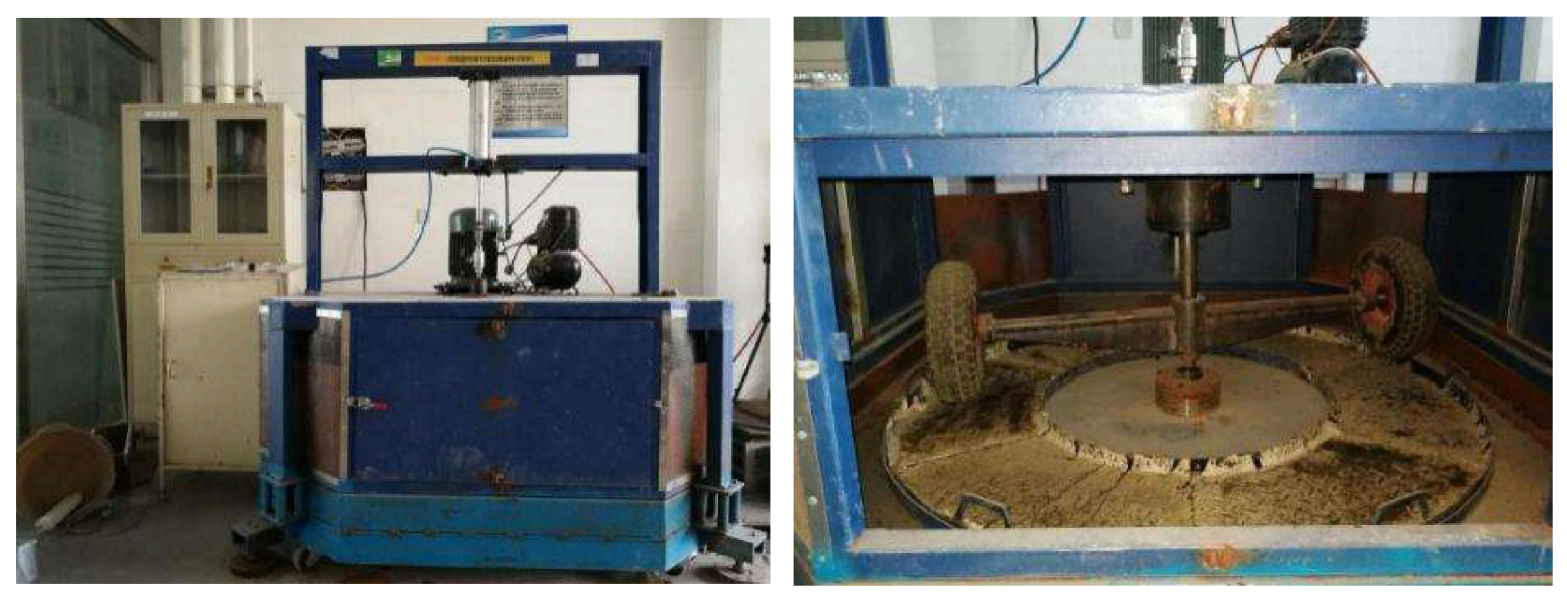
Accelerated abrasion test apparatus for road marking paints.

Given the limited wear volume generated by the Taber test, it struggles to replicate the impact and wear caused by vehicle wheels driving at high speeds. Therefore, to more realistically simulate the actual wear degradation of road marking paints in service, an independently developed accelerated wear tester was employed in this research (shown in Figure 3). Both the specimen preparation format and the application procedures were strictly aligned with practical engineering conditions. A counterweight load of 780 N was applied, and the test was configured for 3000 abrasion cycles. The marking thickness and retroreflectivity were evaluated prior to and following the test, with a minimum of five measurement points for thickness and at least three for retroreflectivity.

#### 2.2.3. Chromaticity Test

According to the JT/T 280-2022 standard [43], the chromaticity of the samples was determined using a colorimeter under standard illuminant D65 and a 45/0 illumination geometry. At least three representative locations were randomly selected on each road marking specimen to record the chromaticity coordinates and luminance factor. The final test result for each sample was obtained by calculating the arithmetic mean of these measurements.

#### 2.2.4. Hiding Power Test

According to GB/T 23981.1-2019 [44], the marking paint was uniformly drawn down onto standard black-and-white hiding power charts using a 300-μm film applicator. The paints were allowed to dry completely for 24 h under standard room conditions (23 ± 2 °C, 50% ± 5% RH). Subsequently, three points were randomly selected on the dried film to measure the luminance factors over the black and white areas using a colorimeter. The arithmetic means of these measurements were obtained to calculate the contrast ratio (defined as the ratio of the luminance factor on the black surface to that on the white surface) to evaluate the hiding power.

#### 2.2.5. Stain Resistance Test

Stain resistance testing was performed according to GB/T 9780-2013 [45] using fly ash as the contaminant medium. The fly ash was thoroughly mixed with water at a 1:1 mass ratio to formulate a soiling suspension. This suspension was uniformly brushed onto the prepared marking specimens using a soft brush (25–50 mm wide) in alternating transverse and longitudinal directions. The panels were dried for 2 h under standard environmental conditions, rinsed, and subsequently dried for 24 h under standard conditions to complete a single cycle. Following five successive cycles, the luminance factor of the test panels was determined. Ultimately, the stain resistance is represented by the percentage decrease in the luminance factor.

#### 2.2.6. Retroreflectivity Measurement

Retroreflectivity testing of the markings was conducted using a portable retroreflectometer according to the JT/T 612-2004 standard [46]. To ensure a stable working condition, the device was preheated for at least 10 min. Zeroing was executed using the equipped black standard block to eliminate background noise like dark current, followed by a calibration step using the supplied reference board to guarantee reading accuracy and repeatability. For the measurement, the instrument was positioned flat against the marking surface, parallel to the traffic direction. It was ensured that the measurement aperture was completely sealed by the marking surface to prevent any test light leakage and to shield the sensor from external stray light. The standard illumination and observation geometry consisted of an entrance angle (*β*_1_) of 88.76° (*β*_2_ = 0°), an observation angle of 1.05°, and maximum aperture angles of 0.33° for both the light source and the receiver.

#### 2.2.7. Skid Resistance Test

According to GB/T 24717-2009 [47] and referring to the pendulum tester method for pavement friction coefficient in JTG 3450-2019 (T 0964-2008) [48], a pendulum friction tester was employed to assess the wet skid resistance of the coating. The measurement principle equates the loss of potential energy of the pendulum to the frictional work performed by a rubber slider moving across the wetted marking surface. The test result is expressed as the Pendulum Test Value (PTV), which is the standard-defined term according to EN 13036-4:2011 [49]. It should be noted that the PTV is numerically equivalent to the British Pendulum Number (BPN) used in some national standards. Before testing, the marking paint was applied to a standard base plate, simulating actual construction processes. Following the curing period, five test points were selected. Upon wetting the surface, the pendulum was released freely from the horizontal position, and the pointer reading was recorded. Five replicate measurements were conducted at each point, and their arithmetic mean was recorded as the local PTV. Finally, the skid resistance of the marking specimen was obtained by dividing the average PTV of the five points by 100.

#### 2.2.8. UV Accelerated Weathering Test

According to GB/T 14522-2008 [50], the UV accelerated weathering test simulates day-and-night exposure in natural environments via alternating cycles of UV radiation and condensation. The irradiation phase utilized fluorescent UV lamps (UVB-313) to replicate daytime solar radiation, maintaining a black panel temperature of 60 ± 3 °C for 8 h. Conversely, the condensation phase simulated nighttime dew by heating the bottom water pan to produce vapor, allowing condensation to form on the specimen surfaces in the dark at a black panel temperature of 50 ± 3 °C for 4 h. A single test cycle lasted 12 h by alternating these two distinct phases. The lamp irradiance was precisely set to 0.63 W/m^2^ at 313 nm. In this research, total weathering periods of 7, 14, and 21 days were selected, equating to 14, 28, and 42 complete cycles, respectively.

#### 2.2.9. VOC Emission Test

Based on the methodological principles of small emission chambers and the specific application features of road marking paints, a closed-chamber test system was specifically designed for measuring VOC emissions. The marking paint was uniformly applied to a 1.0 m × 1.0 m standard asbestos-cement board, strictly following the practical construction process. The coated board was then placed horizontally in a fume hood at room temperature for 15 min until a surface-dry state was achieved. To replicate the continuous release of VOCs under still air conditions and eliminate interference from background ambient VOCs, a 1.0 m × 1.0 m × 1.0 m cubic sealed testing chamber was fabricated from 5-mm-thick commercial corrugated cardboard. To reduce the physical adsorption of VOCs onto the cardboard, the inner walls were lined with aluminum foil to provide an inert surface. All chamber joints were sealed with PVC tape to ensure complete airtightness throughout the testing procedure. Finally, VOC concentrations were measured using an LDARtools portable VOC detector provided by Beijing Dechuang Keyi Technology Co., Ltd. (Beijing, China).

## 3. Results and Discussion

### 3.1. Formulation Design of Waterborne Acrylic Road Marking Paints by Orthogonal Experimental Design

#### 3.1.1. Selection of Factors and Levels for the Orthogonal Experiment

The P/B ratio fundamentally governs the microstructure of the cured coating through its relationship to the PVC—the volume fraction of pigments and fillers in the total solids. The CPVC represents the pigment concentration at which the pigment particles are packed as closely as possible and the binder is exactly sufficient to fill the interstitial voids. Below the CPVC, the binder adequately wets all pigment and filler particles, forming a continuous polymeric matrix that envelops the dispersed solid phase; the coating exhibits optimal mechanical integrity, flexibility, and barrier properties. Above the CPVC, the binder becomes insufficient to fill all voids between particles, leading to the formation of air pockets within the film, reduced film density, and a transition from a continuous to a discontinuous film structure. This transition is accompanied by abrupt changes in mechanical properties, including reduced tensile strength, increased permeability, and diminished scrub resistance. In this study, P/B ratio was selected as the primary formulation variable for orthogonal optimization. This choice was made for practical reasons: (i) P/B can be directly calculated from formulation weights without requiring density data for each component; (ii) P/B is the most commonly used parameter in industrial coating formulation practice for rapid screening and optimization; and (iii) P/B is widely employed in the road marking paint literature. However, we fully recognize that PVC is the theoretically more rigorous parameter. Where possible, we have related our P/B-based optimization to the PVC/CPVC framework in the discussion. Titanium dioxide plays a dual role in the coating system: as the primary functional pigment responsible for opacity and whiteness, and as a reinforcing filler that influences mechanical properties. From an optical perspective, rutile TiO_2_ possesses an exceptionally high refractive index, creating a substantial refractive index mismatch with the acrylic binder. This mismatch results in strong Mie scattering of incident visible light, which is the physical origin of the high hiding power and luminance factor. From a mechanical perspective, well-dispersed TiO_2_ particles act as rigid inclusions within the polymer matrix. Under abrasive wear, these hard particles function as micro-obstacles that deflect and dissipate the energy of abrasive contacts, thereby protecting the softer polymer binder from excessive removal. However, the reinforcing effect is highly dependent on dispersion quality. GCC functions primarily as an extender filler that reduces formulation cost while simultaneously contributing to mechanical reinforcement through a space-filling mechanism. The film formation of waterborne acrylic coatings proceeds through a sequence of well-established stages: (i) water evaporation and particle concentration, (ii) particle packing and deformation, (iii) coalescence of deformed particles, and (iv) interdiffusion of polymer chains across particle boundaries to form a continuous, entangled polymer network. The quality and completeness of this film formation process critically determine the final mechanical properties, optical clarity, and durability of the coating. The coalescing agent plays an indispensable role in this process by temporarily plasticizing the acrylic polymer, thereby reducing its glass transition temperature (Tg) and MFFT [27]. This plasticization enables the polymer particles to deform and coalesce efficiently at ambient temperatures, forming a continuous film without the need for elevated curing temperatures. After film formation is complete, the coalescing agent gradually evaporates from the film, allowing the polymer to regain its original Tg and the associated mechanical strength and hardness. The dosage of the coalescing agent must be carefully optimized. Based on these considerations, four factors were selected for the orthogonal experimental design: P/B ratio (A), TiO_2_ content (B), GCC content (C), and coalescing agent dosage (D). This orthogonal design was implemented to systematically optimize and rapidly screen these variables, ascertaining their respective impact weights on paint performance to achieve an optimally formulated waterborne acrylic road marking paint. An L16 (4^5^) orthogonal array (16 tests, 5 columns, 4 levels each) was adopted, with the specific factors and levels outlined in Table 5. In this context, the amounts of pigment and filler are represented by their mass fractions in the total paint. In standard coating formulation practice, the dosage of coalescing agent is conventionally calculated based on the binder (resin/emulsion) solids content, as the coalescing agent functions by plasticizing the polymer particles to facilitate film formation. However, in this study, the coalescing agent dosage was expressed as a mass fraction based on total paint solids for practical formulation and experimental design reasons: (i) to maintain consistency with the definition of other formulation variables (P/B ratio, TiO_2_ content, and GCC content), which were all based on total formulation mass; (ii) to simplify the weighing and preparation of the 16 orthogonal experimental formulations; and (iii) preliminary trials confirmed that a dosage range of 1–4% of total paint solids was sufficient to achieve complete film formation at ambient temperature for the Type 538 acrylic emulsion. For reference, the corresponding coalescing agent dosage range expressed as a percentage of binder solids is approximately 4–16%, given the P/B ratio range of 2.0:1 to 3.2:1 investigated in this study (calculated as 1–4% of total solids divided by the binder volume fraction).

#### 3.1.2. Performance Test Results of Various Formulations from Orthogonal Tests

In view of the common in-service degradation modes of road marking paints, abrasion resistance, opacity, chromaticity, and stain resistance were specifically chosen as the core performance indices [19,23]. Subsequently, the waterborne acrylic road marking paint, optimized via the orthogonal experimental design, was subjected to additional performance evaluations, including freeze–thaw stability. The test results for the paints formulated according to the orthogonal experiment are detailed in Table 6.

#### 3.1.3. Analysis of Orthogonal Test Results

The experimental results in Table 6 were subjected to a range analysis, with the outcomes detailed in Table 7. Herein, *k*_1_, *k*_2_, *k*_3_, and *k*_4_ denote the mean values of the performance indices when a certain factor is set at levels 1, 2, 3, and 4, respectively. The range *R* is the difference between the maximum and minimum k values within the same factor. A larger *R* value indicates a more substantial influence of that factor on the evaluated index. Finally, according to the performance requirements for each indicator, the optimal level of each factor was selected and subsequently combined to determine the optimal formulation.

(1) Abrasion resistance

**Figure 4:**
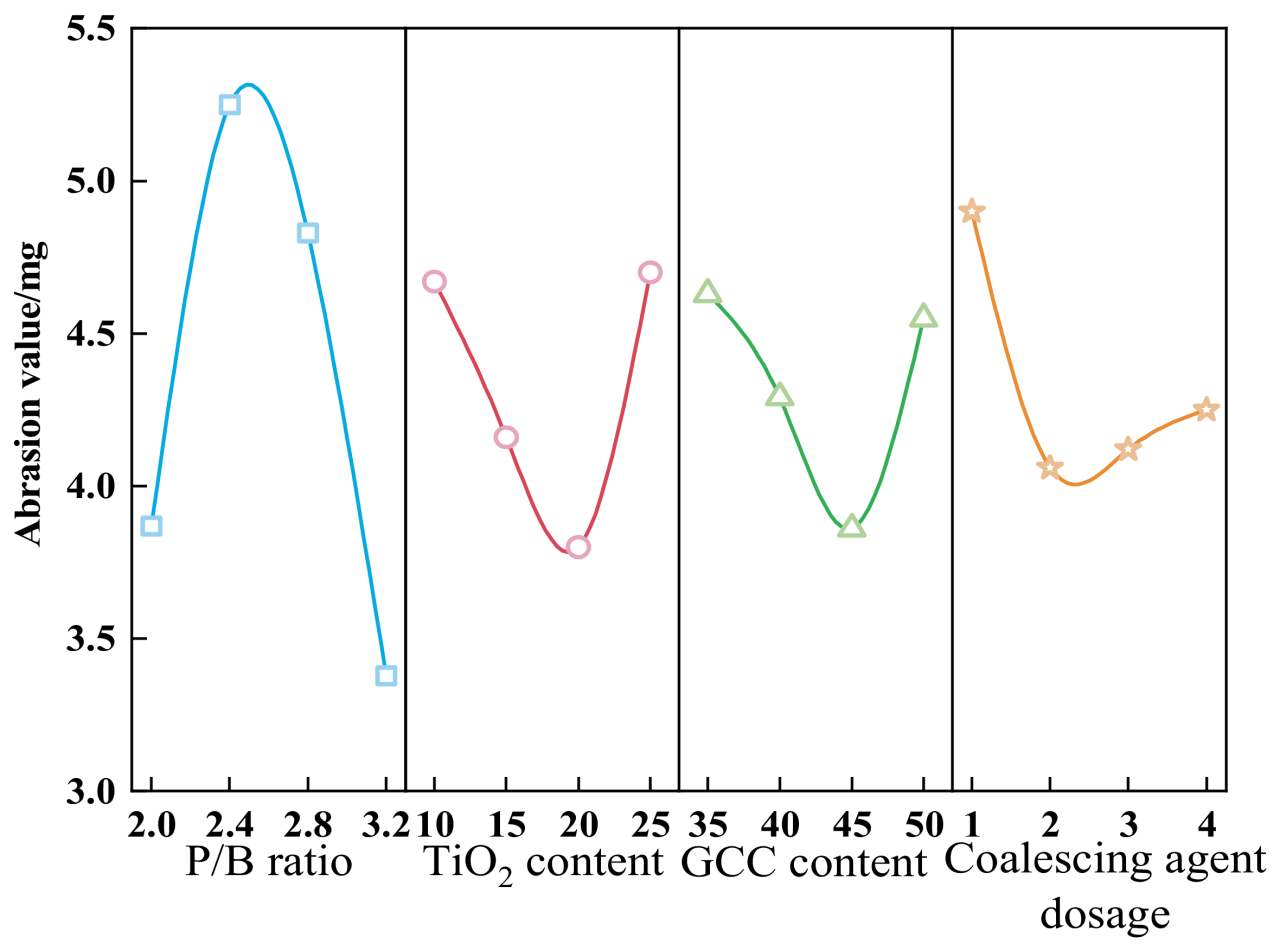
Influence of various factors on the abrasion value.

As shown in Table 7 and Figure 4, the abrasion mass loss of the prepared marking paints was significantly lower than the 40 mg limit specified in relevant standards (e.g., GB/T 16311-2024 [51] or JT/T 280-2022 [43]), demonstrating excellent abrasion resistance. Among the various factors affecting abrasion resistance, the P/B ratio exhibited the most significant impact. When the P/B ratio was 3.2, the mass loss of the coating reached its minimum value. It indicates that the pigments and fillers exhibit optimal compatibility with the binder system, ensuring their stable dispersion within the waterborne acrylic resin system and thereby effectively enhancing the abrasion resistance. With the increase in the dosages of TiO_2_ and GCC, the abrasion mass loss of the marking coatings showed an overall trend of initially decreasing and subsequently increasing. The relatively high hardness of the pigments and fillers plays a positive role in improving the wear resistance. However, when the TiO_2_ content reached 25%, and the GCC content reached 50%, local excesses of pigments and fillers tended to agglomerate due to uneven dispersion, thereby exerting an adverse effect on the abrasion resistance. It should be noted that the contribution of pigment and filler hardness to abrasion resistance is not absolute; if the pigment and filler concentration exceeds the CPVC of the resin system, the binding capacity of the resin to the solid particles will decrease. It results in a loose structure that paradoxically accelerates wear. The degree of impact of each factor on the abrasion resistance, in descending order, was A > B > D > C. Based on the mean value analysis, the optimal combination for abrasion resistance was A_4_B_3_D_2_C_3_ (see Table 7 for level definitions).

(2) Hiding power

**Figure 5:**
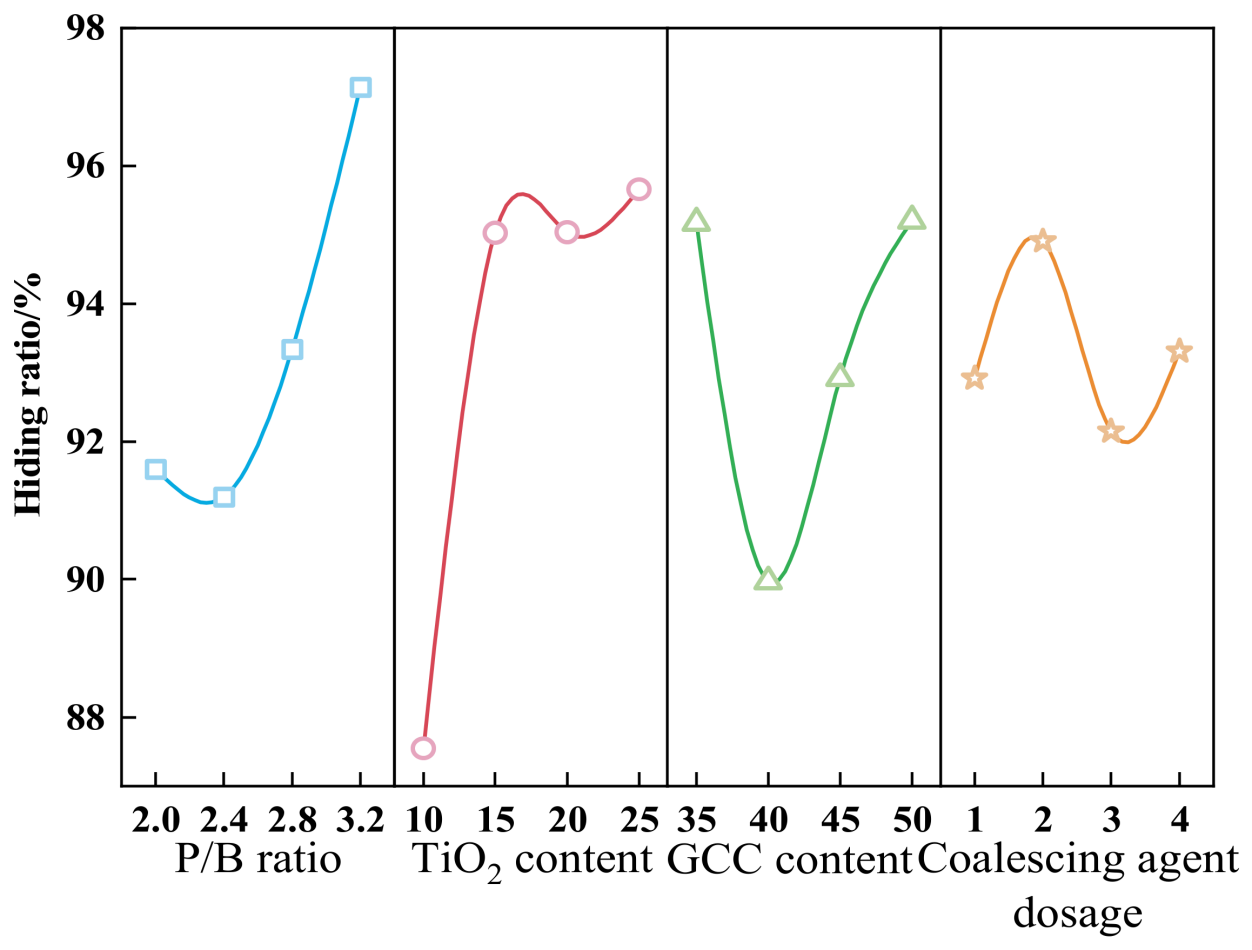
Influence of various factors on the hiding power.

Fundamentally, the hiding power of marking paints depends on the absorption and scattering capabilities of the coating film towards light, with the scattering contribution playing a dominant role. According to optical principles, the scattering capability of pigments and fillers towards white light is closely related to their refractive indices. A greater ratio between the refractive index of the pigments/fillers and that of the binder yields a stronger light scattering capability, thereby resulting in better hiding power. The TiO_2_ utilized in this study possesses a refractive index of 2.78, making it the white pigment with the most outstanding hiding power. Consequently, as the content of TiO_2_ in the system increases, the hiding power exhibits a continuous upward trend. Simultaneously, with the increase in the P/B ratio, the hiding power also displayed an overall increasing trend. It should be pointed out that the hiding power of marking paints serves only as one of the reference factors for evaluating application efficiency, and higher values do not necessarily indicate superiority. In paint formulation design, while an increase in the dosage of pigments and fillers improves the hiding power, the PVC of the system increases accordingly. Once it exceeds the CPVC, the performance will undergo an abrupt change and exhibit a downward trend [19]. Under such conditions, the pigment particles are insufficiently wetted, leading to the formation of voids within the pigment-binder matrix. Consequently, the coating film transitions from a continuous to a discontinuous state, which severely compromises its mechanical properties and durability. Therefore, the hiding power must be evaluated in conjunction with other indicators, such as abrasion resistance, requiring a comprehensive balance to determine the optimal formulation design. As shown in Table 7 and Figure 5, TiO_2_ content (Factor B) had the most pronounced effect on hiding power, followed by the P/B ratio, GCC content, and coalescing agent dosage. The optimal combination for hiding power alone was B_4_A_4_C_4_D_2_, corresponding to the maximum levels of TiO_2_ (25%), GCC (50%), and P/B ratio (3.2), with a coalescing agent dosage of 2%.

(3) Luminance factor

**Figure 6:**
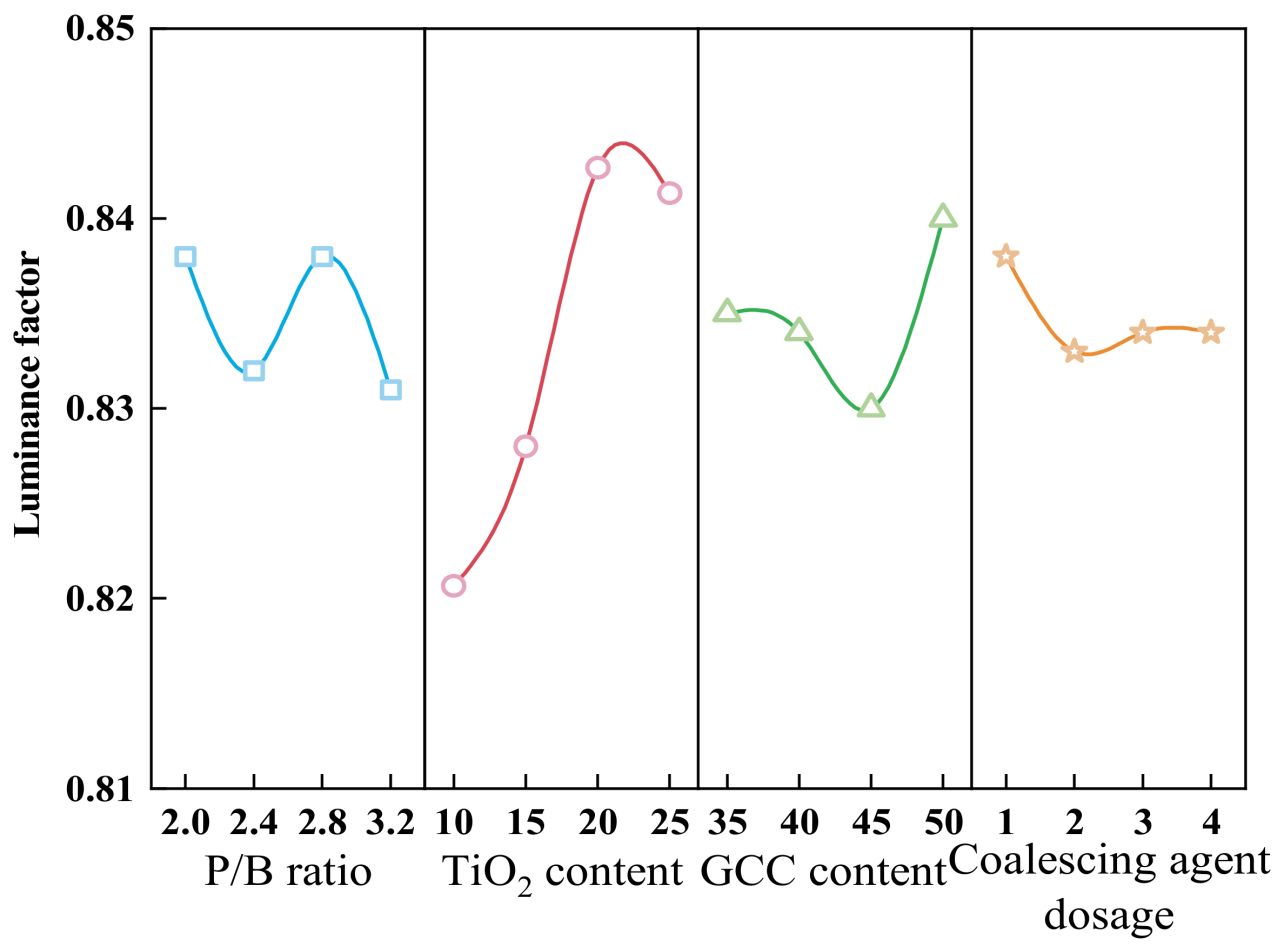
Influence of various factors on the luminance factor.

Colorimetric performance is a core technical indicator for evaluating the daytime visibility of road markings. Generally, a surface with higher reflectivity possesses a larger luminance factor. This indicates that, under identical illumination conditions, the markings exhibit higher surface luminance, which assists drivers in perceiving and recognizing traffic marking information at an earlier stage. In accordance with relevant standards, a colorimeter was employed in this study to systematically characterize the colorimetric properties of the paints across different experimental formulations. As shown in Table 7 and Figure 6, the TiO_2_ dosage exerted the most significant impact on the luminance factor. From the perspective of optical mechanisms, rutile TiO_2_ possesses an exceptionally high refractive index [52]. The substantial refractive index difference between the TiO_2_ and the binder endows the pigment with intense scattering capability. This effectively enhances the reflection efficiency towards incident light, thereby significantly improving its whiteness and hiding power. In white road marking systems, the luminance factor predominantly relies on the reflection and scattering of visible light by pigments and fillers. Due to its superior light-scattering performance, TiO_2_ acts as the critical component governing the luminance factor. This perfectly aligns with the range analysis conclusion, which identifies TiO2 as the primary influencing factor. Based on the range analysis, the impact significance of each factor on the colorimetric properties followed the descending order: B > C > A > D. Using colorimetric performance as the evaluation indicator, the optimal formulation combination was determined to be B_3_C_4_A_3_D_1_. Specifically, the paint achieves optimal colorimetric properties when the TiO_2_ content was 20%, the GCC content was 50%, the P/B ratio was 2.8, and the coalescing agent content was 1%. In this optimal combination, both the TiO_2_ dosage and the P/B ratio are at appropriate levels rather than their maximum extremes, which further validates the rationality of the formulation design.

(4) Stain resistance

**Figure 7:**
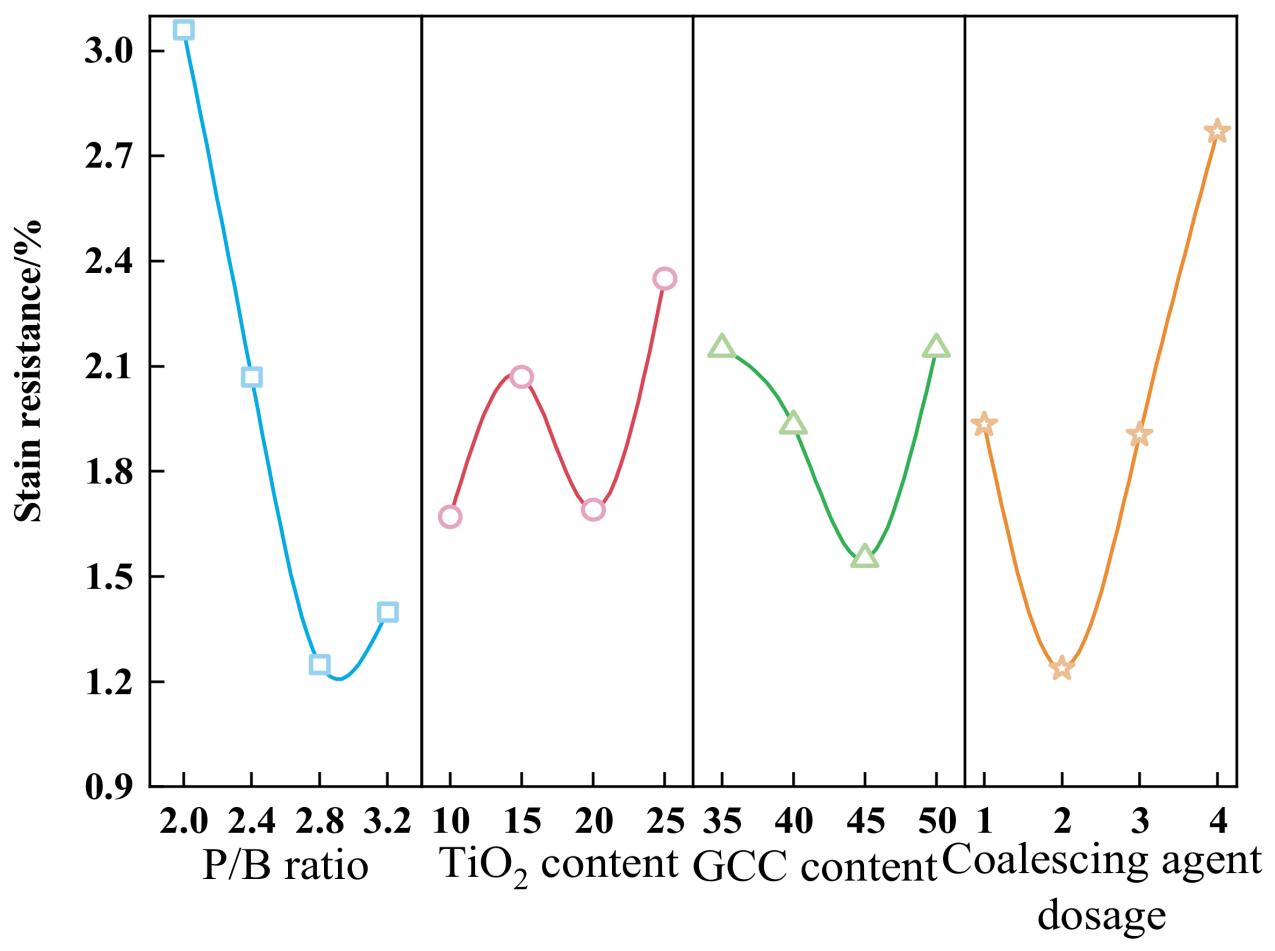
Influence of various factors on the stain resistance.

The range analysis of the orthogonal experimental results (see Table 7 and Figure 7) indicates that the coalescing agent dosage was the primary factor affecting the stain resistance of the coating, exerting the most significant influence on the test results. As a crucial additive in the waterborne acrylic emulsion system, the coalescing agent promotes the deformation and coalescence of polymer particles during the drying process by softening them, thereby forming a uniform, continuous, and dense coating film. Its impact on stain resistance is primarily manifested in the following two aspects. First, the coalescing agent can significantly reduce the minimum MFFT of the waterborne polymer, enabling the emulsion to form a film efficiently at ambient temperatures. Second, the coalescing agent helps form a dense coating film by eliminating the internal micro-cracks inherently present in films prepared from hard emulsions, thereby enhancing the coating’s resistance to stains. When the coalescing agent dosage accounts for 2% of the total solid content, it can effectively promote sufficient coalescence and crosslinking of latex particles. It leads to the formation of a smooth, flat, and dense marking coating, which significantly reduces the physical adhesion tendency of stains onto the surface. An insufficient dosage leads to discontinuous film formation, resulting in a rough surface with residual capillary micro-pores that serve as channels for stain adsorption and penetration. Conversely, an excessive dosage may leave residual plasticized polymers on the coating surface, causing it to become overly soft and paradoxically deteriorating the stain resistance. The range magnitude of the impact of each factor on the stain resistance followed the order: D > A > B > C. Using stain resistance as the evaluation indicator, the optimal design combination was D_2_A_3_B_1_C_3_. That is, the optimal stain resistance was achieved when the P/B ratio was 2.8, the TiO_2_ content was 10%, the GCC content was 45%, and the coalescing agent dosage was 2%. In this optimal combination, the TiO_2_ dosage was relatively moderate, while the coalescing agent was selected at the second-highest level, which further validates the dominant role of the coalescing agent in enhancing the stain resistance.

#### 3.1.4. Formulation Optimization Based on a Comprehensive Weighted Scoring Method

To ensure the scientific validity and effectiveness of the comprehensive optimization analysis, a comprehensive weighted scoring method was employed in this study to convert the multiple performance indicators from the orthogonal experiment into a single comprehensive index [53,54]. The core of this method lies in introducing weights to distinguish the relative importance of each indicator. Its key aspect is ensuring the rationality of the scoring criteria and the scientific validity of the weight design.

First, an evaluation matrix was established based on the orthogonal experimental results (Table 6). Let n be the number of orthogonal experimental schemes (*n* = 16), and m be the number of evaluation indicators (*m* = 4, namely abrasion resistance, hiding power, colorimetric properties, and stain resistance). Then, the matrix *X* = (*x*_ij_)*_n_*_×*m*_ serves as the evaluation matrix of the scheme set against the indicator set, as shown in Equation (1).

(1) $$X=\left[\begin{array}{llll} 5.60 & 88.13 & 0.82 & 2.36\\ 2.88 & 90.96 & 0.83 & 2.02\\ 3.16 & 91.31 & 0.85 & 2.44\\ 3.84 & 96.00 & 0.85 & 5.41\\ 4.80 & 81.11 & 0.81 & 2.35\\ 5.80 & 94.32 & 0.83 & 3.60\\ 5.00 & 93.82 & 0.86 & 1.82\\ 5.40 & 95.57 & 0.83 & 0.50\\ 4.10 & 86.58 & 0.82 & 1.53\\ 5.18 & 96.60 & 0.83 & 0.91\\ 3.79 & 98.69 & 0.85 & 0.87\\ 6.24 & 91.48 & 0.86 & 1.70\\ 4.17 & 94.40 & 0.82 & 0.44\\ 2.77 & 98.24 & 0.81 & 1.74\\ 3.26 & 96.34 & 0.84 & 1.64\\ 3.33 & 99.59 & 0.85 & 1.78 \end{array}\right],$$

Subsequently, the evaluation matrix was normalized to eliminate the influence of dimensional and order-of-magnitude differences among the indices. Among the four evaluation indicators, abrasion resistance (mass loss) and stain resistance (reduction rate of the luminance factor) are negative indicators, meaning that a smaller value denotes superior performance. Therefore, they were processed using the corresponding normalization formula (see Equation (2)). Conversely, hiding power and colorimetric properties (luminance factor) are positive indicators, where a larger value signifies better performance; thus, they were calculated using the normalization formula for positive indicators (see Equation (3)). Following the normalization process, a dimensionless evaluation matrix *Z* = (*z*_ij_)*_n_*_×*m*_ was obtained, as expressed in Equation (4).

(2) $$z_{\mathrm{ij}}=\frac{{\left(x_{j}\right)}_{\max}-x_{\mathrm{ij}}}{{\left(x_{j}\right)}_{\max}-{\left(x_{j}\right)}_{\min}},$$

(3) $$z_{\mathrm{ij}}=\frac{x_{\mathrm{ij}}-{\left(x_{j}\right)}_{\min}}{{\left(x_{j}\right)}_{\max}-{\left(x_{j}\right)}_{\min}},$$

(4) $$Z=\left[\begin{array}{cccc} 0.18 & 0.38 & 0.23 & 0.61\\ 0.97 & 0.53 & 0.53 & 0.68\\ 0.89 & 0.55 & 0.78 & 0.60\\ 0.69 & 0.81 & 0.80 & 0.00\\ 0.41 & 0.00 & 0.00 & 0.62\\ 0.13 & 0.71 & 0.38 & 0.36\\ 0.36 & 0.69 & 1.00 & 0.72\\ 0.24 & 0.78 & 0.51 & 0.99\\ 0.62 & 0.30 & 0.25 & 0.78\\ 0.31 & 0.84 & 0.44 & 0.91\\ 0.71 & 0.95 & 0.72 & 0.91\\ 0.00 & 0.56 & 0.90 & 0.75\\ 0.60 & 0.72 & 0.25 & 1.00\\ 1.00 & 0.93 & 0.19 & 0.74\\ 0.86 & 0.82 & 0.62 & 0.76\\ 0.84 & 1.00 & 0.81 & 0.73 \end{array}\right],$$

In a multi-indicator comprehensive evaluation, the rational determination of weights is crucial for ensuring the reliability of the evaluation results. To guarantee the rationality and reliability of the comprehensive scoring, this study employed a combined weighting approach, integrating both subjective and objective weighting methods, to determine the final weights of the evaluation indicators.

The subjective weight vector was established based on two complementary considerations. First, the practical application requirements of waterborne road marking paints prioritize abrasion resistance, hiding power, and luminance factor as the primary performance criteria, as these directly determine the service life, application efficiency, and daytime visibility of the markings. Stain resistance, while important, is considered a secondary criterion because marking contamination can be partially mitigated through regular maintenance. Second, the range analysis results from the orthogonal experiment (Table 7) quantitatively confirmed that the P/B ratio and TiO_2_ content exert the most significant influences on the core performance indicators, further supporting the assignment of higher weights to abrasion resistance, hiding power, and luminance factor. Accordingly, the subjective weights were designated as *α* = [0.3, 0.3, 0.3, 0.1]^T^ for abrasion resistance, hiding power, colorimetric properties, and stain resistance, respectively.

Subsequently, the entropy weight method (EWM) was utilized to calculate the objective weights of the evaluation indicators. As an objective weighting technique based on the principle of information entropy, the EWM’s core concept is to measure the dispersion degree of each evaluation indicator using information entropy. A smaller information entropy value indicates greater variability of the indicator and a larger amount of information provided, thereby resulting in a higher assigned weight. The EWM determines the objective weights through successive steps, including decision matrix construction, data normalization, information entropy calculation, and difference coefficient normalization. The calculation formula for the entropy value is presented in Equation (5), where it is stipulated that *p*_ij_ ln*p*_ij_ = 0 when *p*_ij_ = 0. By defining the difference coefficient of the indicator *d*_j_ as 1-*E*_j_, the objective weight *β*_j_ of the *j*-th indicator can be calculated using Equation (6). Based on these principles, the objective weight vector for abrasion resistance, hiding power, colorimetric properties, and stain resistance, calculated via the EWM, is *β* = [0.35, 0.19, 0.31, 0.16]^T^.

(5) $$E_{j}=-K\sum_{i=1}^{n}p_{\mathrm{ij}}\ln p_{\mathrm{ij}}\left(K=1/\ln n\right),$$

(6) $$\beta_{j}=d_{j}/\sum_{k=1}^{m}d_{k},$$

Once both the subjective and objective weights were established, a preference coefficient *μ* (0 < *μ* < 1) was introduced to perform a linear weighted combination of the two, thereby determining the comprehensive weight of the *j*-th indicator (see Equation (7)). By integrating this preference coefficient *μ*, the combined weighting method achieves an optimal coordination between subjective experience and objective information. It balances practical engineering demands with statistical data characteristics, rendering the scoring criteria more scientifically rational and the result analysis more reliable. A value of *μ* = 0.5 was selected to endow subjective engineering judgment and objective data-driven information equal importance. This balanced approach is widely adopted in multi-criteria comprehensive evaluation studies, as it avoids the potential bias inherent in purely subjective weighting (e.g., over-reliance on individual expert experience) while also mitigating the limitations of purely objective weighting (e.g., neglect of domain-specific engineering knowledge). The combination of subjective and objective weights enhances the stability and reliability of the evaluation results. According to this combined weighting model, with *μ* set to 0.5, the comprehensive weight vector for the indicators was calculated as *w* = [0.32, 0.24, 0.31, 0.13]^T^. Furthermore, utilizing the comprehensive weighted scoring formula (see Equation (8)), the comprehensive score vector for the orthogonal experimental schemes was derived as *f* = [0.302, 0.692, 0.734, 0.665, 0.213, 0.376, 0.680, 0.551, 0.449, 0.555, 0.797, 0.507, 0.572, 0.703, 0.764, 0.855]^T^. This score vector precisely represents the multi-indicator comprehensive scores for all tested schemes.

(7) $$w_{j}=\mu \alpha_{j}+\left(1-\mu \right)\beta_{j},$$

(8) $$f_{i}=\sum_{j=1}^{m}z_{\mathrm{ij}}w_{j},$$

To further investigate the differential impacts of various factors on the comprehensive scores and the varying degrees of influence at different levels, a range analysis was performed on the comprehensive score vector (see Table 8).

The range analysis results indicate that the magnitude of influence of each factor on the comprehensive weighted score follows the descending order: B > A > D > C. Notably, the TiO_2_ dosage exerts the most significant impact on the comprehensive performance. Based on a comparative analysis of the mean values at different levels for each factor, the optimal combination, when evaluated by the comprehensive weighted score, is determined to be B_3_A_4_D_2_C_4_. Specifically, the optimal formulation consists of a TiO_2_ content of 20%, a P/B ratio of 3.2, a coalescing agent dosage of 2%, and a GCC content of 50%. This optimal proportion demonstrates excellent consistency with the optimal combinations derived from the individual indicators, thereby further validating the rationality of the selected formulation. Given the P/B ratio of 3.2:1 (i.e., pigment/filler solids constitute approximately 76.2% of total solids, and binder solids constitute approximately 23.8%), the equivalent coalescing agent dosage based on binder solids is calculated as 8.4% on binder solids. For the optimized formulation, the pigment and filler volume fractions relative to total solids volume yield a PVC of approximately 62.5%. For reference, the CPVC of the acrylic binder system with this pigment/filler combination was estimated to be approximately 65~70% based on the oil absorption values of the pigments and fillers. The optimized PVC of 62.5% lies slightly below the estimated CPVC, indicating that the binder is sufficient to wet all pigment and filler particles and form a continuous film without excessive void formation. The pH of the optimized formulation was measured using a calibrated pH meter at 23 ± 2 °C and was found to be 8.7, which is within the recommended range for waterborne acrylic systems (typically 8.0~9.5) to ensure emulsion stability and optimal dispersant performance.

#### 3.1.5. Performance Testing of the Optimal Formulation

Comprehensive performance testing of the optimized formulation was conducted according to JT/T 280-2022 [43], with all results summarized in Table 9. Notably, the optimized paint substantially exceeded the standard requirements across all key metrics (particularly in abrasion resistance, hiding power, and non-tack drying time) while also demonstrating superior resistance to water and alkali exposure (72 h without degradation, compared to the 24 h standard requirement). These results fully verify the efficacy and reliability of the orthogonal optimization strategy.

### 3.2. Retroreflective Performance

**Figure 8:**
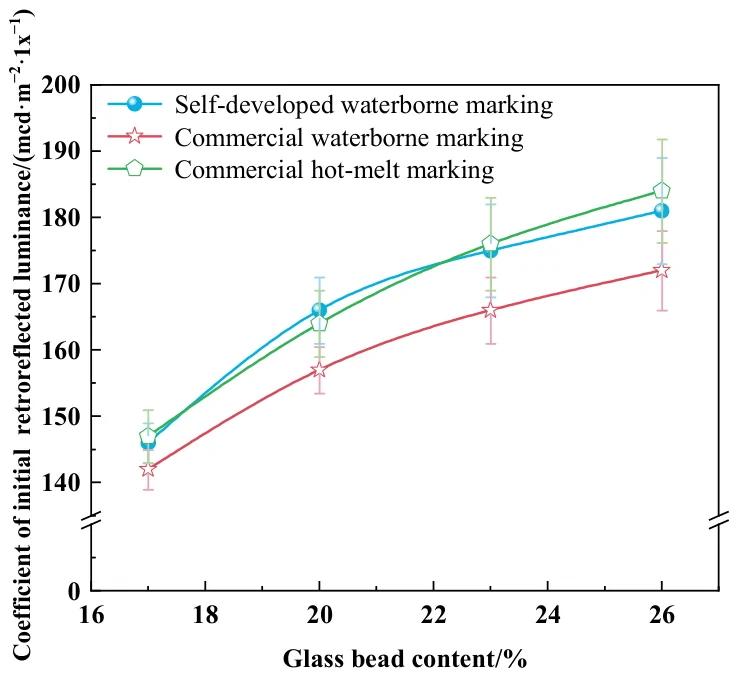
Effect of different glass bead content on the coefficient of retroreflected luminance of road markings.

The visibility of road traffic markings is a crucial performance indicator for their evaluation, while the coefficient of retroreflected luminance serves as the core quantitative parameter of marking visibility. This coefficient directly determines the recognizability of markings under low-visibility conditions, such as nighttime, rain, or fog, and is of vital significance to ensuring nighttime driving safety. Research indicates that the application of drop-on glass beads is a critical technical measure to enhance the nighttime visibility of road markings [59,60]. Incident light is refracted within the glass beads and reflected parallel back to the light source, creating a retroreflective effect that enables drivers to clearly identify the position and alignment of markings at night. Additionally, high-quality glass microbeads can absorb and dissipate external impact energy, thereby protecting the marking coating to a certain extent and delaying its wear and aging processes. For the comparative evaluation, a commercial waterborne marking paint and a commercial hot-melt (thermoplastic) marking paint were selected as benchmarks. Hot-melt paint was chosen as a benchmark because it represents the dominant technology for high-traffic highways and major roads, offering exceptional durability and retroreflectivity. Demonstrating that our waterborne formulation can achieve comparable or superior performance to hot-melt paint, while offering substantial environmental benefits, represents a more significant scientific contribution than comparison against solvent-borne paints, whose environmental disadvantages are already well established. The commercial hot-melt paint was applied as a sprayed hot-melt type at a temperature of 180~200 °C, with a controlled film thickness of 1.5~2.0 mm, consistent with standard practice for highway markings. Solvent-borne and cold plastic (MMA-based) paints were not included as benchmarks because: (i) solvent-borne paints are increasingly being phased out due to strict VOC regulations and their inferior environmental performance is well documented; and (ii) cold plastic paints are two-component reactive systems with fundamentally different chemistry and application requirements, making direct comparison with our single-component waterborne acrylic system less straightforward. Therefore, in accordance with the GB/T 16311-2024 standard [51], the initial coefficient of retroreflected luminance was tested for the self-developed waterborne marking (optimal formulation) derived from orthogonal experimental optimization, a commercial waterborne marking, and a commercial hot-melt marking, respectively. The test results are illustrated in Figure 8. The glass bead dosages expressed as percentages in Figure 8 represent the mass fraction of glass beads relative to the total mass of the wet paint used in laboratory specimen preparation. For the retroreflectivity measurements, specimens were prepared by spray application onto asbestos-cement boards (300 mm × 300 mm) to simulate actual field conditions, achieving a dry film thickness of 1.5~2.0 mm. The 20% dosage corresponds to an approximate surface application rate of 350~400 g/m^2^, which falls within the typical recommended range of 300~400 g/m^2^ for drop-on glass bead application. The 17% dosage represents the lower end of this range, while 23% and 26% represent higher-than-recommended loadings.

The results demonstrate that the glass bead dosage significantly impacts the initial coefficient of retroreflected luminance across different types of markings. At a lower glass bead dosage (17%), the initial coefficients for all three markings failed to meet the minimum requirement of 150 mcd·m^−2^·lx^−1^ specified by the GB/T 16311-2024 standard [51]. The underlying reason is that, at this dosage, the number of glass beads distributed per unit area on the marking coating is insufficient. Consequently, incident light cannot be focused and efficiently reflected toward the source along its original path by an adequate number of glass beads. The limited reflective cross-section results in an overall low retroreflective efficiency. As the glass bead dosage increased from 17% to 20%, the coefficients of retroreflected luminance for the three markings exhibit the most rapid growth trend, with increments of 13.7%, 10.6%, and 11.6%, respectively. When the dosage was further increased beyond 20%, the growth trend for all three markings tended to plateau. This phenomenon can be attributed to the fact that once the glass bead application rate exceeds a critical threshold, the beads begin to accumulate and overlap on the coating surface. An excessively high distribution density causes a shadowing effect between adjacent glass beads; consequently, incident light is lost after undergoing multiple ineffective reflections among the microbeads, paradoxically reducing the effective reflectivity. Concurrently, overly dense application reduces the ideal embedment depth of the glass beads within the coating (typically around 60% of the bead diameter), further impairing the retroreflective performance of the markings [28,29].

In comparison across different marking types, the initial coefficient of retroreflected luminance of the self-developed waterborne marking was significantly superior to that of the commercial waterborne marking at all dosages, and was generally comparable to that of the commercial hot-melt marking. This reflects the effective embedment depth and uniform distribution of the glass beads within the paint film, which are governed by the rheological properties of the wet paint and the application conditions. Given the relatively high cost of glass beads, and comprehensively considering both the enhancement efficiency of retroreflective performance and the economic cost of materials, the optimal glass bead dosage for this system is recommended to be 20%.

### 3.3. Abrasion Wear Resistance

#### 3.3.1. Taber Test Results

**Figure 9:**
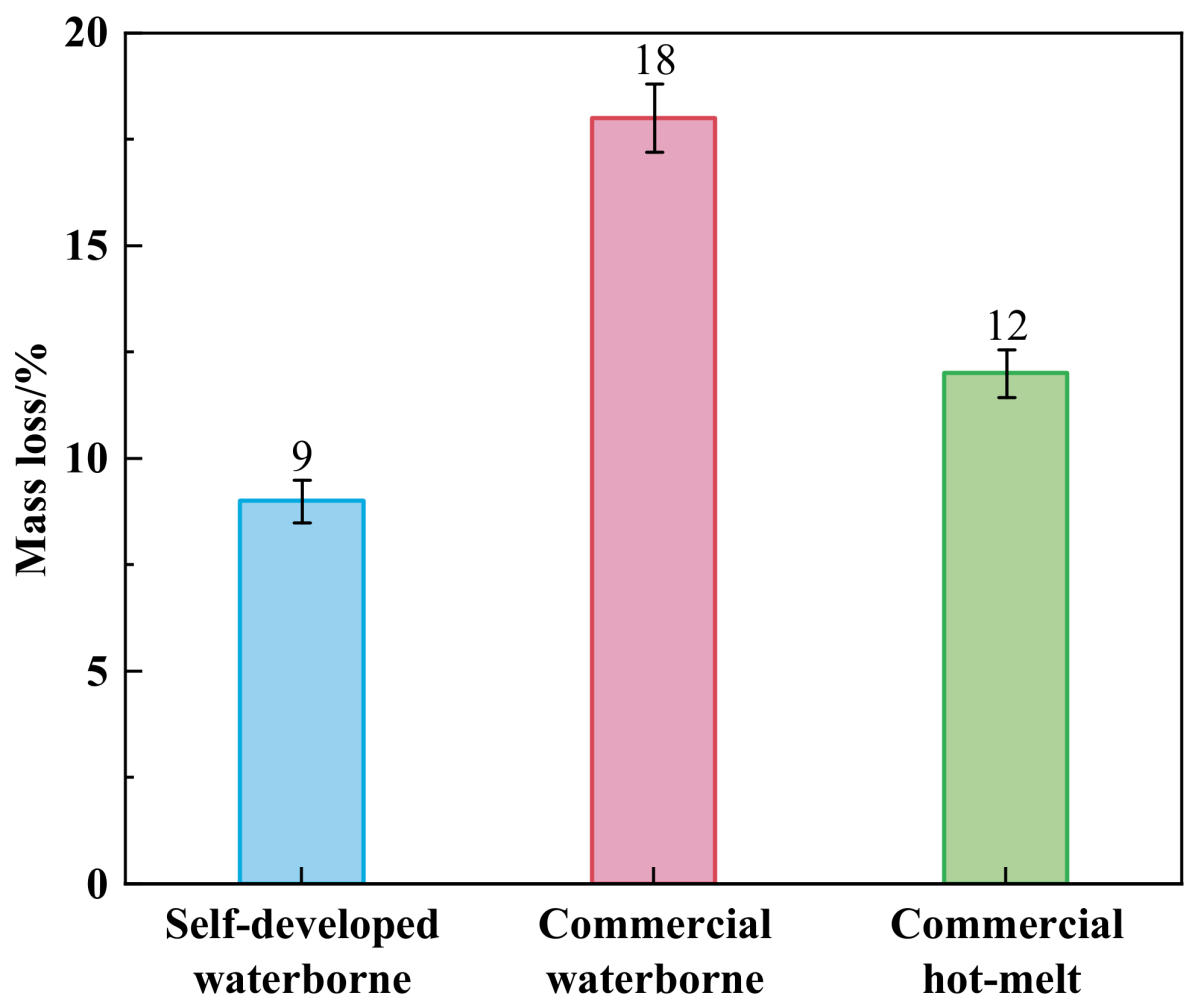
Mass loss results obtained after the Taber abrasion test.

Figure 9 presents the mass loss results of the three marking paints following the Taber abrasion test, which was performed on bare coating specimens without glass beads (premix or drop-on) for all three marking types to evaluate the intrinsic abrasion resistance of the cured film. The data indicate that the mass loss of the self-developed waterborne marking coating was 9 mg, compared to 18 mg for the commercial waterborne marking and 12 mg for the commercial hot-melt marking. The mass loss is expressed in milligrams (mg), as specified in GB/T 1768-2006 [42].

Notably, the self-developed waterborne coating exhibited the lowest mass loss value. A smaller mass loss signifies a stronger capacity of the material to resist abrasive wear. Consequently, the self-developed waterborne marking coating demonstrates the best abrasion resistance among the three evaluated materials. This superior performance is primarily attributed to the excellent compatibility and dispersion stability between the waterborne acrylic resin binder and the solid pigments and fillers [19]. Such properties facilitate the formation of a stable abrasion equilibrium layer during the wear process, thereby effectively inhibiting the large-scale detachment of the coating. Furthermore, the optimized P/B ratio and coalescing agent dosage in the formulation contribute to increasing the film density, which further enhances the mechanical wear resistance.

#### 3.3.2. Accelerated Abrasion Test Results

**Figure 10:**
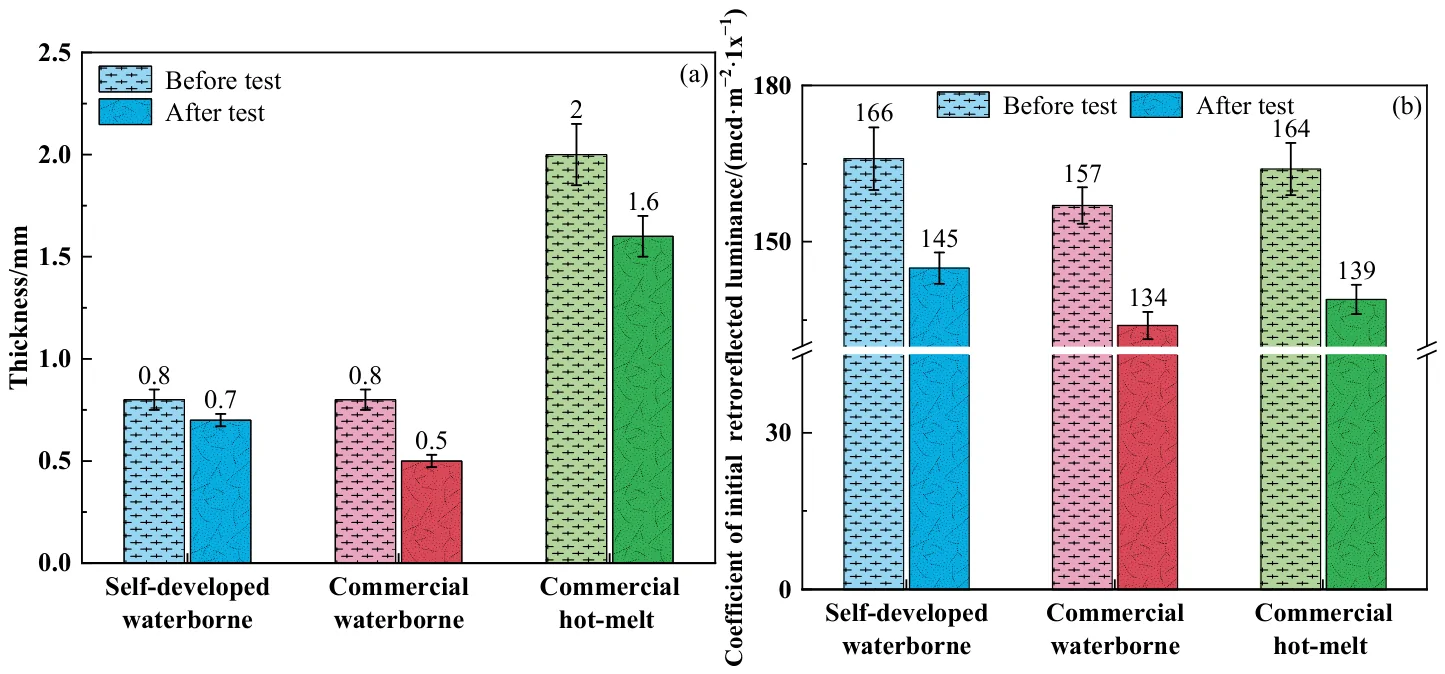
Test results of (**a**) marking thickness and (**b**) coefficient of retroreflected luminance before and after the accelerated abrasion test.

Figure 10 presents the test results for the marking thickness and the coefficient of retroreflected luminance before and after the accelerated abrasion test. The results indicate that the thickness variation of the three types of markings was not significant after 3000 cycles of accelerated abrasion. Notably, the self-developed waterborne marking coating exhibited the minimum thickness loss of only 0.1 mm, demonstrating excellent abrasion resistance. Furthermore, the self-developed waterborne marking displayed the highest retention rate of the coefficient of retroreflected luminance (87.3%) after abrasion, outperforming both the commercial waterborne (85.4%) and hot-melt (84.8%) marking coatings. This verifies its potential as a high-performance road marking material with superior durability in both wear resistance and retroreflective maintenance. It is worth noting that the trend of the loss in the coefficient of retroreflected luminance is essentially consistent with that of the thickness loss. This is primarily because, as the marking coating gradually thins due to abrasion, the anchoring effect of the coating on the glass beads is weakened, leading to an increased detachment and loss rate of the glass beads, which subsequently causes a continuous decay in the retroreflective performance of the markings [3].

### 3.4. Weathering Resistance

**Figure 11:**
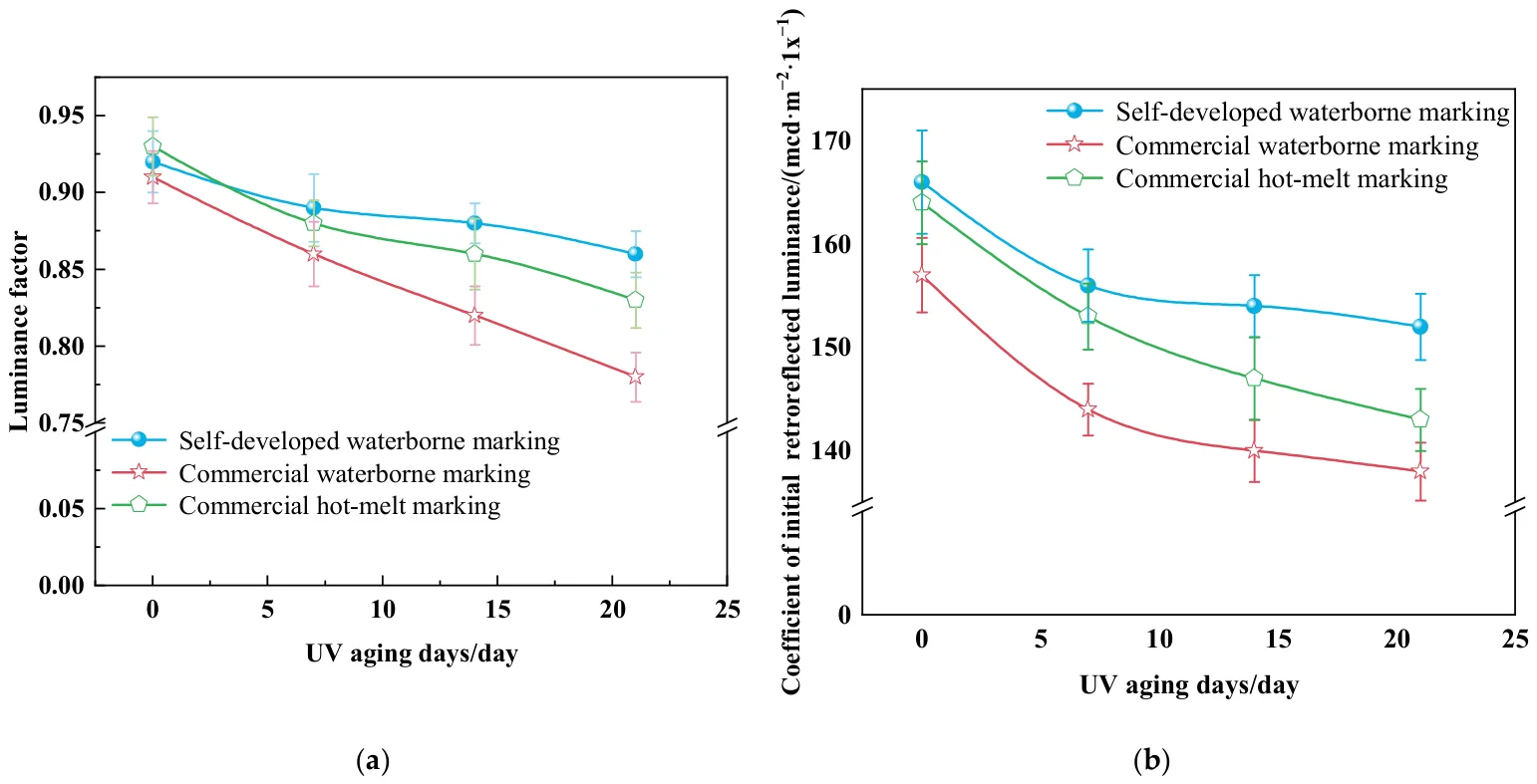
Variation of (**a**) luminance factor and (**b**) coefficient of retroreflected luminance of road marking paint with accelerated aging days.

Chromaticity and retroreflective performance are two key indices for characterizing the weathering resistance of waterborne acrylic road marking coatings. Chromaticity is primarily evaluated by the luminance factor, while retroreflective performance is quantified by the coefficient of retroreflected luminance. The variations in the luminance factor and the coefficient of retroreflected luminance for the three sets of marking samples over different aging periods are presented in Figure 11.

According to the JT/T 280-2022 standard, the luminance factor of white marking coatings must be no less than 0.80. Test results confirm that all three sample groups meet this specification. As shown in Figure 11, both the luminance factor and the coefficient of retroreflected luminance of the three coatings exhibited varying degrees of decay during accelerated aging. Regarding chromaticity, the rate of decline in the luminance factor, from fastest to slowest, was as follows: commercial waterborne marking > commercial hot-melt marking > self-developed waterborne marking. These results indicate that the self-developed waterborne marking coating outperforms the two comparative materials in chromaticity stability, thereby exhibiting superior weathering resistance. Regarding the decay of retroreflective performance, the self-developed waterborne marking maintained the highest coefficient of retroreflected luminance across all aging cycles, followed by the commercial hot-melt marking, while the commercial waterborne marking exhibits the lowest values. Notably, both the self-developed and commercial waterborne markings showed a slowing decay trend after 7 days of aging. It is hypothesized that during the initial aging stage, the resin on the coating surface degrades preferentially, leading to the partial detachment of embedded glass beads and a sharp drop in the coefficient of retroreflected luminance. As aging continues, the degradation reaction gradually migrates into the coating interior, and the surface becomes covered by a degraded layer; consequently, the rate of further photochemical reactions becomes diffusion-controlled, leading to a moderated decay in retroreflective performance. In contrast, the coefficient of retroreflected luminance of the commercial hot-melt marking exhibited a monotonic decreasing trend throughout the test cycle without a significant slowing trend, which may be related to the compactness of its film-forming system and its specific degradation kinetics.

Furthermore, during the accelerated aging test, significant cracking and peeling were observed on the surface of the commercial waterborne marking after 21 days, whereas the self-developed waterborne marking remained intact with no signs of cracking or delamination [24]. This macro-scale failure is highly consistent with the aforementioned retroreflective test results. Surface cracking destroys the anchoring interface of the glass beads, preventing the maintenance of an effective embedment depth, which in turn accelerates the decay of the coefficient of retroreflected luminance. The occurrence of cracking and peeling further indicates that the film-forming resin of the commercial waterborne marking undergoes severe chain scission and crosslinking under photo-oxidation, leading to the accumulation of internal stress until failure. In conclusion, the self-developed waterborne marking coating demonstrates superior weathering resistance compared to the commercial products in terms of chromaticity retention, retroreflective stability, and surface integrity.

### 3.5. Skid Resistance

**Figure 12:**
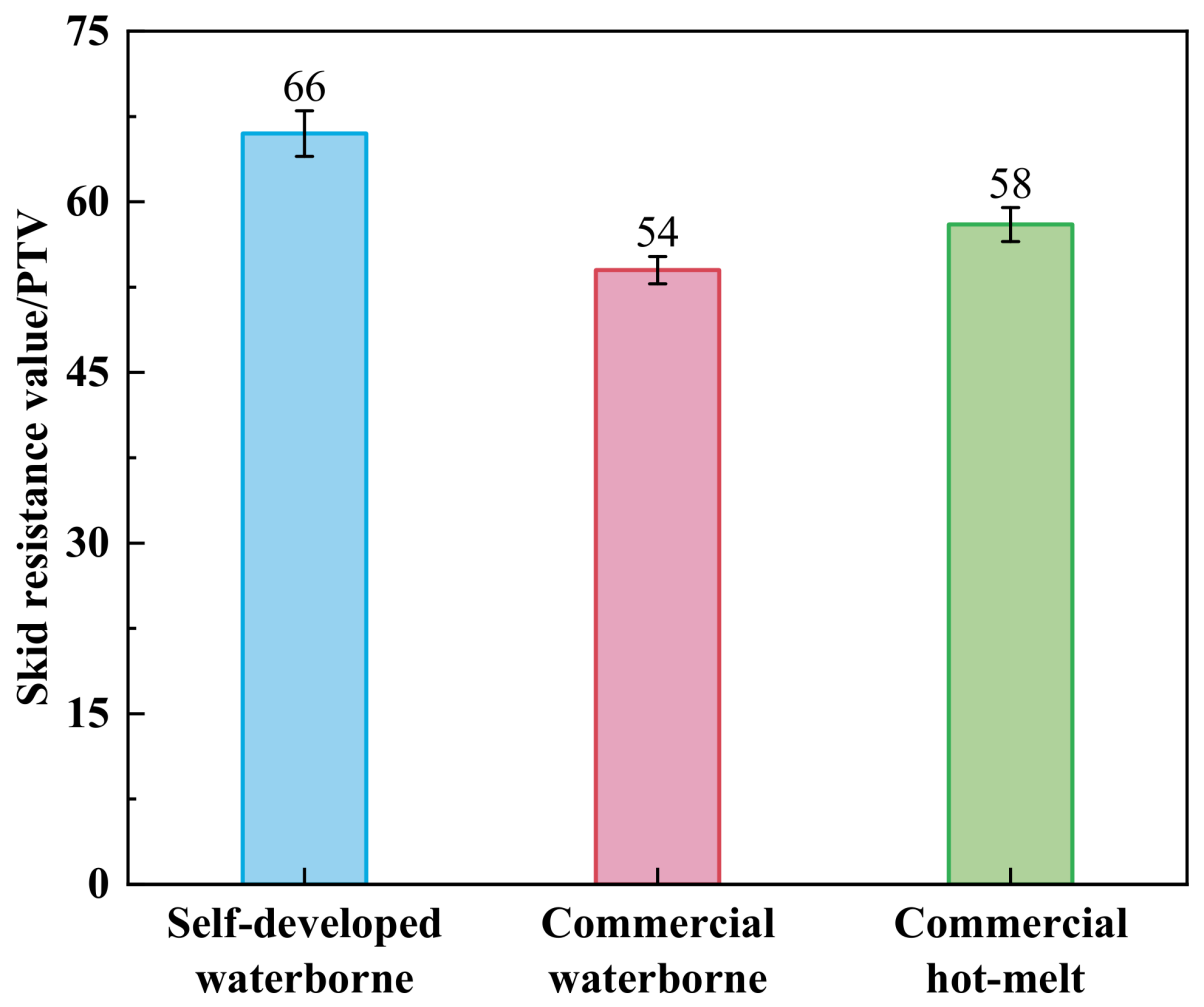
Skid resistance test results of the road markings.

According to the GB 51038-2015 [61] and GB/T 16311-2024 standards [51], the skid resistance of road traffic markings shall be no less than 45 PTV. A higher PTV indicates a greater coefficient of friction on the marking surface, implying richer micro-texture and superior skid resistance under low-adhesion conditions such as wet pavement [4]. Figure 12 presents the skid resistance test results for the self-developed waterborne marking, the commercial waterborne marking, and the commercial hot-melt marking. The results indicate that all three marking coatings satisfy the specification requirements, while exhibiting significant differences in skid resistance: the self-developed waterborne marking performed the best, followed by the commercial hot-melt marking, with the commercial waterborne marking exhibiting the lowest values. The superior skid resistance of the self-developed waterborne marking can be explained by three key factors. First, the moderate thickness of the waterborne coating enables the formation of surface morphology similar to the texture characteristics of the asphalt pavement, thereby effectively increasing the mechanical interlocking between the tires and the markings. Second, the high viscosity of the coating helps maintain the structural integrity of the film during application, providing sufficient anchoring depth for the glass beads. Moreover, the self-developed waterborne marking utilizes acrylic polymers as the binder; as the water evaporates, the polymer matrix crosslinks with the glass beads to form a dense 3D network structure, which enhances the interfacial bonding strength between the glass beads and the coating, rendering the beads less prone to detachment under traffic loads. Third, the glass beads form uniform and appropriately protruding micro-structures within the coating, which further enriches the micro-texture of the marking surface and significantly improves the skid resistance in wet environments. The combined effect of these factors ensures that the self-developed waterborne marking exhibits better overall skid resistance than the commercial waterborne and hot-melt alternatives.

### 3.6. VOC Emissions

**Figure 13:**
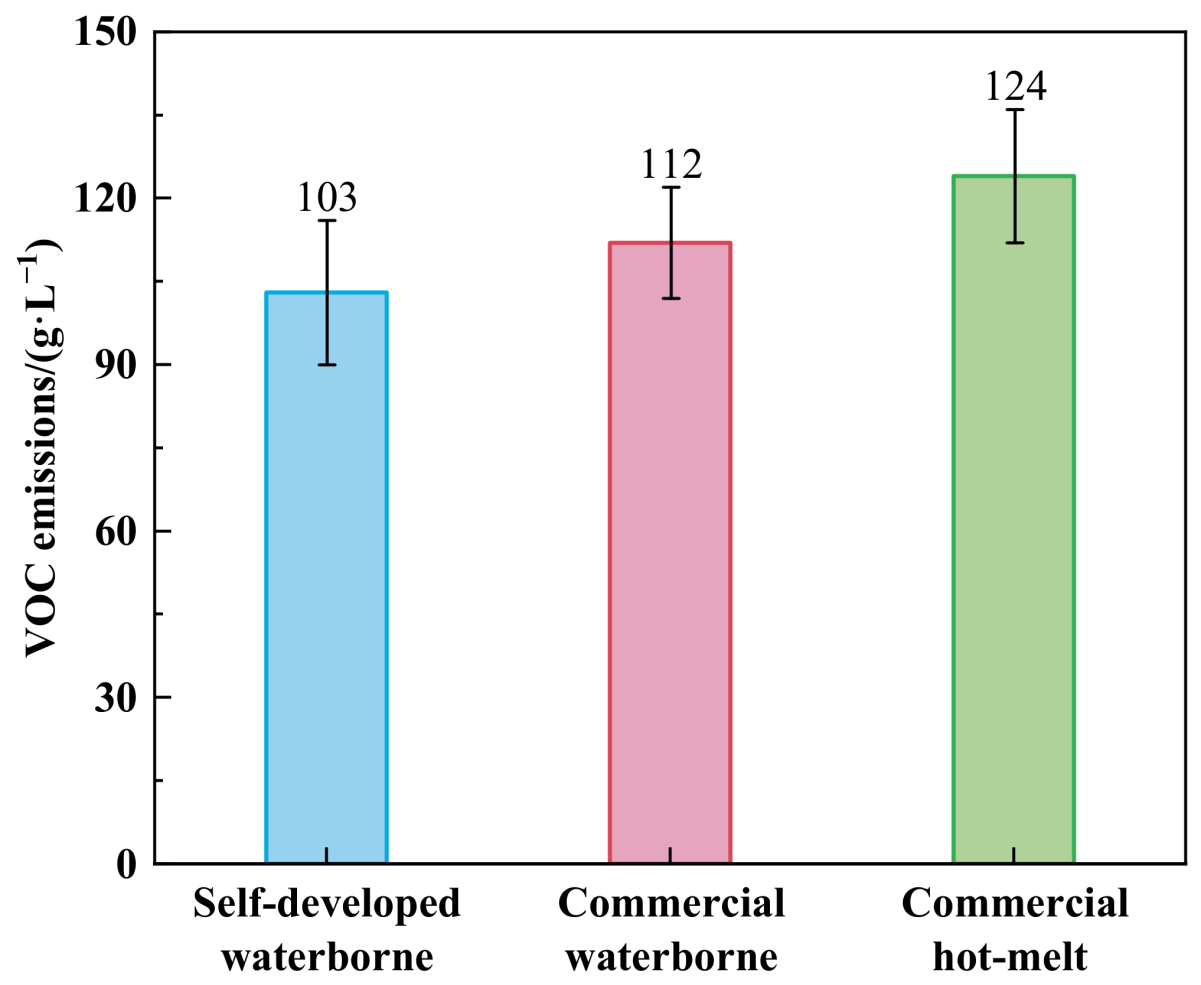
VOC emissions from different road markings.

VOC emissions were measured using a self-designed sealed chamber system to evaluate the environmental performance of the developed paint. As shown in Figure 13, the self-developed waterborne marking coating exhibited the lowest VOC emission among the tested samples, with a measured value of 103 g/L, compared to 112 g/L for the commercial waterborne marking coating. The difference of approximately 9 g/L (~8%) was consistently observed across replicate measurements and falls within the expected range for waterborne acrylic systems. For the commercial hot-melt paint, which is a solid powder at room temperature, VOC emissions are typically negligible during storage and handling. According to the JT/T 280-2022 standard [43], the VOC content of hot-melt road marking paints is limited to ≤50 g/kg. However, during the high-temperature application process (typically 180~200 °C), thermal degradation of organic components (including C5 petroleum resins, polyethylene wax, and plasticizers) can release volatile decomposition products such as low-molecular-weight hydrocarbons and oxygenated compounds [62,63]. Nevertheless, even accounting for such thermal degradation, the VOC emissions of hot-melt paints remain substantially lower than those of solvent-borne systems and are generally considered to be “negligible” or “zero-VOC” in the literature. The self-developed waterborne paint (103 g/L) thus offers a meaningful environmental advantage over both commercial waterborne and hot-melt alternatives, with emissions well below the typical limits for waterborne systems [13,64].

### 3.7. Field Application Verification of the Self-Developed Waterborne Road Marking Paint

To verify the practical construction and field performance of the self-developed waterborne marking paint, this study conducted a field application validation on an operational section of the Hegang-Dalian Expressway (G11) within Liaoning Province, China. The experimental segment is located at K1151 + 900 on the southbound lane of the Hegang-Dalian Expressway, with a total length of 152 m and a total marking area of 73 m^2^, utilizing a single-marking application scheme. According to the traffic statistics provided by the Liaoning Provincial Transportation Department, the Annual Average Daily Traffic (AADT) for this section is approximately 28,000~32,000 vehicles per day, with a heavy vehicle (truck and bus) proportion of approximately 35~40%.

#### 3.7.1. Construction Technology and Equipment

Given the small construction volume of the test section, a hand-pushed high-pressure airless spraying device was adopted for the construction work. In cold-spray road marking operations, high-pressure airless spraying is a widely used technique for ambient-temperature road marking applications. This equipment utilizes a gasoline-engine-driven plunger pump to apply high pressure to the paint, which causes the paint to undergo instantaneous atomization upon releasing hydraulic pressure through the nozzle and then be sprayed onto the road surface. It features uniform film formation, easily controllable coating thickness, and high construction efficiency, making it suitable for the application of various traffic markings, including lines, arrows, and patterns.

#### 3.7.2. Construction Environment Control

**Figure 14:**
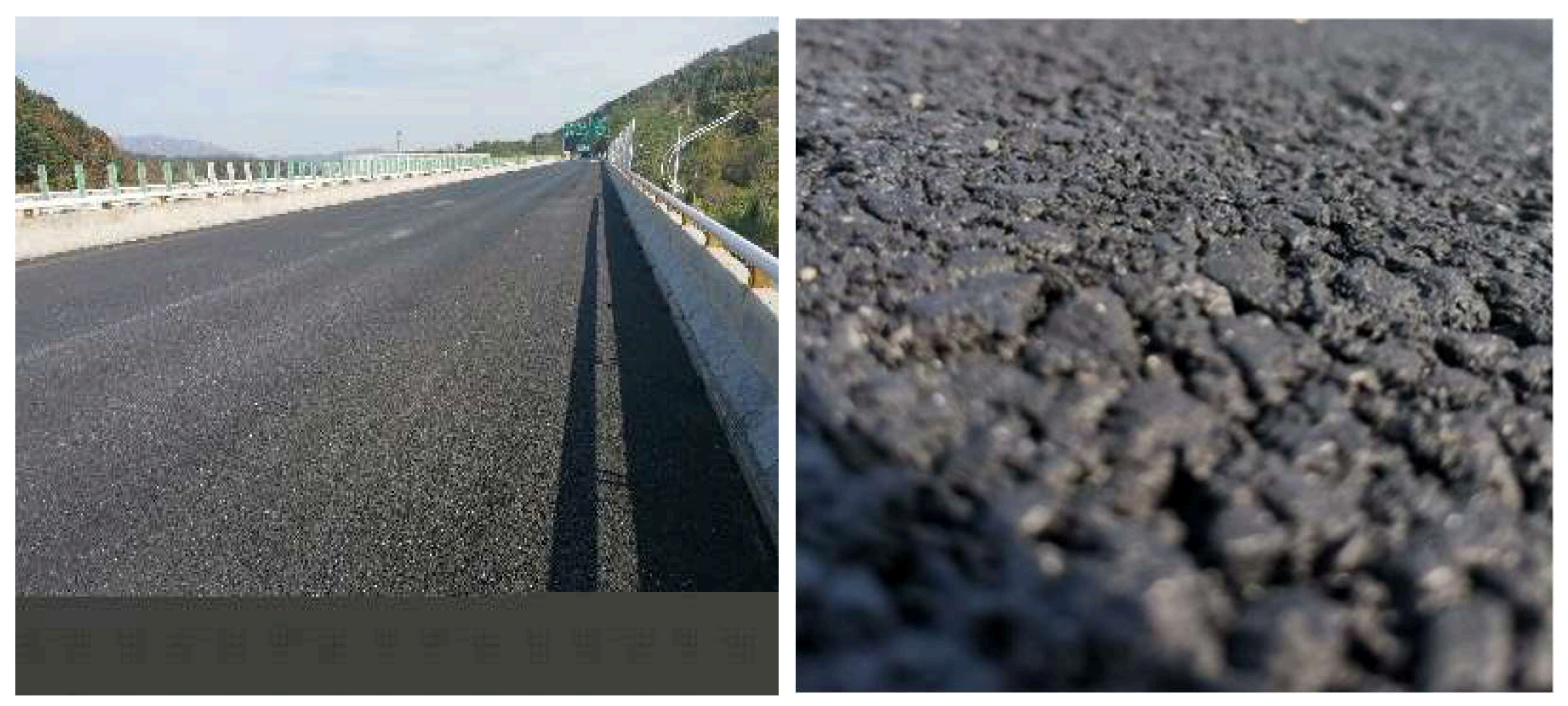
Road surface of the test section before marking application.

The film-forming performance of waterborne acrylic road marking paints exhibits strong dependence on construction environmental conditions. The drying mechanism of waterborne coatings is dominated by water evaporation and latex particle coalescence, and their film-forming rate is significantly governed by ambient temperature and relative humidity. Previous studies have shown that waterborne road marking coatings can achieve a dry-to-traffic time of ≤10 min (allowing low-speed vehicle traffic at this stage) under construction conditions of temperature ≥ 10 °C, relative humidity ≤ 85%, and light breeze. Full curing generally requires more than 30 min. In addition, no precipitation should occur within 24 h before and after paint application to prevent water infiltration into the incompletely cured coating. Meanwhile, the road surface must be kept clean and dry, free from loose aggregates, dust, and oil contamination, to ensure effective adhesion between the paint and the road surface. Based on the above theoretical basis, the on-site construction was conducted under outdoor conditions with ambient temperature ≥ 10 °C and relative humidity ≤ 80%. Road surface cleaning and drying were completed before construction (see Figure 14), ensuring the compliance of the construction environment.

#### 3.7.3. Construction Effect and Performance Testing

**Figure 15:**
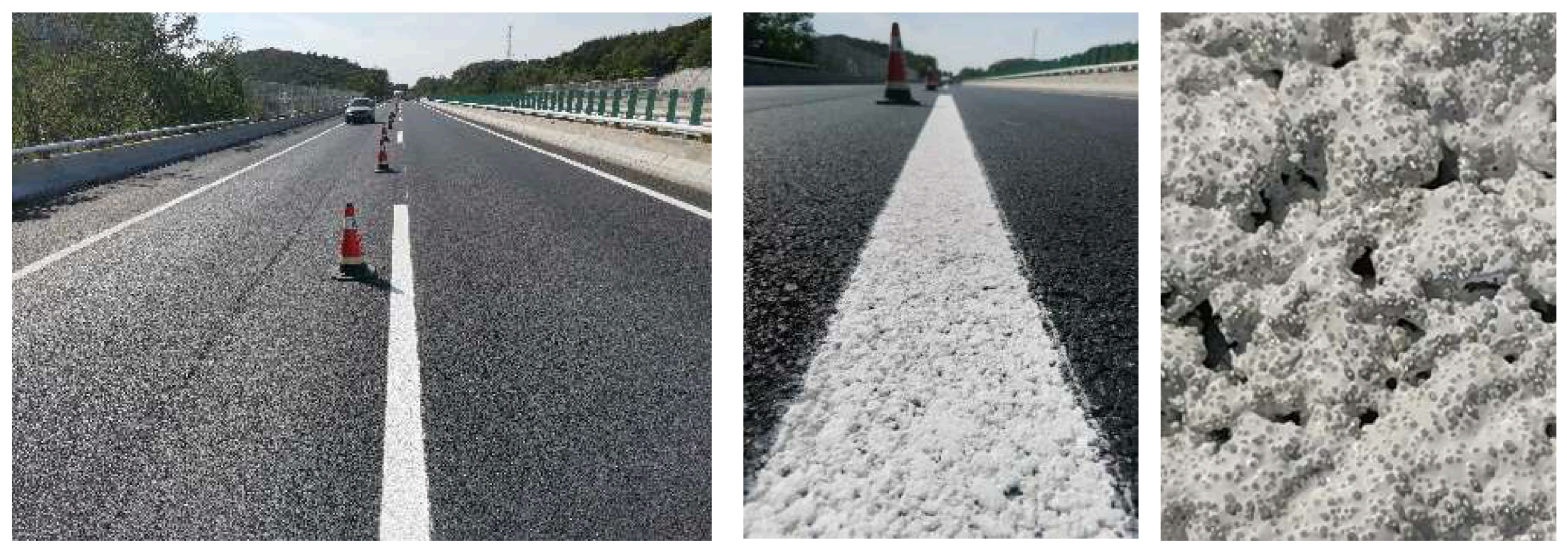
Test section after marking application.

Field observation results after marking application (see Figure 15) demonstrate that the drop-on glass beads form excellent embedding and matching with the paint, exhibiting an ideal settlement and uniform distribution state within the marking film.

Engineering acceptance inspections were conducted on the newly marked test section in accordance with the requirements of GB/T 16311-2024 [51] and JT/T 280-2022 [43], focusing on the on-site detection of initial retroreflective luminance coefficient and marking thickness. According to the acceptance criteria specified in GB/T 16311-2024 [51], the initial retroreflective luminance coefficient of white Type I retroreflective markings shall not be less than 150 mcd·m^−2^·lx^−1^, and the wet film thickness should be in the range of 0.3~0.8 mm. Field measurements at five representative locations yielded an average retroreflective luminance coefficient of 410.6 mcd·m^−2^·lx^−1^ (ranging from 353 to 454 mcd·m^−2^·lx^−1^) and an average marking thickness of 0.578 mm (ranging from 0.52 to 0.64 mm). The measured retroreflectivity substantially exceeds the minimum standard requirement of 150 mcd·m^−2^·lx^−1^ and is considerably higher than the average value of 115.7 mcd·m^−2^·lx^−1^ reported in a recent field study, as well as surpassing typical initial values of 150–250 mcd·m^−2^·lx^−1^. It indicated that the self-developed waterborne marking paint and the selected glass beads possess excellent interfacial compatibility and synergistic retroreflective effect under actual engineering conditions. Meanwhile, the marking thickness also falls within the standard-recommended wet film thickness range. Both key performance indicators meet the requirements of current standards, fully verifying the construction feasibility and technical reliability of the self-developed waterborne acrylic road marking paint in practical engineering applications.

### 3.8. Cost–Performance Analysis and Economic Feasibility

While the preceding sections have demonstrated the superior technical performance of the optimized waterborne acrylic road marking paint, the commercial viability of any formulated product ultimately depends on its cost–performance ratio. This subsection provides a transparent cost analysis and discusses the cost–performance trade-off from both initial material cost and life-cycle perspectives.

#### 3.8.1. Raw Material Cost Estimation

Based on current domestic market prices in China (as of 2025–2026), rutile TiO_2_ is quoted at approximately 13,800–14,800 CNY per ton, while GCC is substantially less expensive at approximately 800–1200 CNY per ton. The waterborne acrylic resin emulsion (Type 538) is priced at approximately 8000–10,000 CNY per ton, and the coalescing agent at approximately 12,000–15,000 CNY per ton. Using these benchmark prices, the estimated raw material cost of our optimized formulation (P/B ratio of 3.2, 20% TiO_2_, 50% GCC, and 2% coalescing agent) is approximately 4200–4800 CNY per ton of paint. For comparison, a typical commercial waterborne paint with lower TiO_2_ content (e.g., 10–12%) would have a raw material cost of approximately 3500–4000 CNY per ton, while a high-performance commercial hot-melt paint would cost approximately 4500–5500 CNY per ton owing to its higher resin and binder content. It should be noted that the 20% TiO_2_ loading in our formulation is within the typical concentration range for water-based road marking paints (10–25% by weight) and is comparable to that of solvent-based systems (15–20%). While this loading is higher than some low-end thermoplastic products (which may contain as little as 5–10% TiO_2_), it is essential to recognize that such low-TiO_2_ formulations often exhibit inferior whiteness, hiding power, and long-term visibility retention, necessitating more frequent repainting and higher life-cycle costs.

#### 3.8.2. Performance Benefits Justifying the TiO_2_ Loading

The elevated TiO_2_ content in our optimized formulation is justified by the substantial performance improvements it delivers across multiple critical metrics:

(1) Luminance factor of 0.86, significantly exceeding the minimum requirement of 0.80 specified in JT/T 280-2022 [43], ensuring superior daytime visibility.
(2) Hiding power of 98%, which minimizes the required coating thickness for complete opacity and reduces material consumption per unit area.
(3) Abrasion mass loss of only 2.1 mg, far below the 40 mg standard limit, indicating exceptional wear resistance that extends service life.
(4) Superior weatherability, with no cracking or peeling observed after 21 days of UV-accelerated aging, whereas commercial waterborne paints exhibited severe surface deterioration under identical conditions.

These performance advantages translate directly into extended service life and reduced maintenance frequency. In road marking applications, the cost of materials is often dwarfed by the cost of application (traffic control, labor, equipment mobilization) and the societal cost of traffic disruptions during repainting operations. A paint that requires less frequent repainting can therefore offer superior life-cycle cost-effectiveness despite a higher initial material cost.

#### 3.8.3. Life-Cycle Cost Perspective

From a life-cycle cost perspective, the superior durability of our developed paint (particularly its outstanding abrasion resistance and weatherability) suggests that the intervals between repainting operations could be extended compared to conventional waterborne or low-TiO_2_ alternatives. While long-term field performance data are still being collected, the accelerated laboratory test results provide strong evidence that the optimized formulation possesses the potential for extended service life. In practice, the extended service life of high-performance markings reduces not only material consumption but also the frequency of traffic disruptions, labor costs, and the environmental footprint associated with repeated application cycles.

#### 3.8.4. Summary of Cost–Performance Assessment

In summary, while the 20% TiO_2_ loading in our optimized formulation represents a higher initial material cost compared to some low-end commercial products, this loading is within the typical range for high-performance waterborne road marking paints and is justified by the substantial and quantifiable performance benefits it delivers. From a life-cycle perspective, the extended service life and reduced maintenance frequency enabled by the superior durability of our paint may offset the higher initial material cost. Furthermore, we have identified several promising directions for future cost optimization, including partial substitution with functional extenders and the incorporation of advanced polymer technologies, which could further enhance the economic competitiveness of the formulation without compromising its technical performance.

## 4. Conclusions

In this study, an eco-friendly waterborne acrylic road marking paint was developed to address the high VOC emissions and limited durability of traditional marking materials. By integrating an orthogonal experimental design with a comprehensive weighted scoring method based on subjective and objective entropy weights, the formulation was systematically optimized. Based on comprehensive laboratory evaluations and field application validation, the main conclusions are drawn as follows:

(1) Formulation optimization

The optimal formulation of the waterborne acrylic road marking paint is determined as a P/B ratio of 3.2, a TiO_2_ dosage of 20%, a GCC dosage of 50%, and a film-forming agent content of 2% based on total solid content. This optimized combination synergistically maximizes wear resistance, hiding power, and luminance factor while maintaining excellent stain resistance.

(2) Retroreflectivity enhancement

The incorporation of surface-applied glass beads significantly impacts the nighttime visibility of the markings. An optimal glass bead dosage of 20% was identified, which effectively maximizes the initial coefficient of retroreflected luminance without causing detrimental overlapping and shading effects between the beads.

(3) Superior durability and mechanical performance

In both Taber and self-developed accelerated abrasion tests, the optimized paint showed the lowest mass loss (9 mg in the Taber test) and thickness loss (only 0.1 mm after 3000 cycles), as well as the highest retroreflectivity retention (87.3%), demonstrating superior resistance to mechanical wear and bead anchorage stability under simulated traffic loading. After 21 days of UV-accelerated aging, the developed paint maintained a luminance factor above 0.80 and exhibited the smallest decrease in both luminance factor and coefficient of retroreflected luminance among all tested paints. No cracking or peeling was observed on its surface, whereas the commercial waterborne paint showed severe cracking after 21 days, confirming the exceptional weatherability of the optimized formulation. The skid resistance of the developed paint also surpassed that of the commercial alternatives.

(4) Significant environmental benefits

The developed paint demonstrates an excellent eco-friendly profile. The VOC emission of the proposed waterborne paint was measured as 103 g/L, which is 8.0% lower than that of commercial waterborne paint. This significant reduction highlights its substantial potential in promoting green and sustainable transportation infrastructure.

(5) Successful field validation

Real-world application on a highway section in Liaoning Province, China, confirmed the practical constructability of the developed paint using hand-pushed high-pressure airless spraying equipment under controlled conditions (temperature ≥ 10 °C, relative humidity ≤ 80%). The measured field coefficient of retroreflected luminance (410.6 mcd·m^−2^·lx^−1^) and wet film thickness (0.578 mm) fully complied with GB/T 16311-2024 requirements [51], validating the laboratory-optimized formulation for practical engineering use.

In summary, the newly developed waterborne acrylic road marking paint achieves a balanced trade-off among mechanical durability, optical performance, weather resistance, and environmental impact. Its low VOC emission, high retroreflectivity, and excellent abrasion resistance make it a promising candidate for replacing conventional solvent-based or hot-melt road marking materials, particularly in regions with strict environmental regulations and high demands for traffic safety. The long-term durability data under real service conditions remain to be collected and will be the focus of our future work. We acknowledge that certain mechanistic aspects were beyond the scope of the current study. These include direct measurements of interfacial adhesion energy between the acrylic binder and GCC/TiO_2_ particles (e.g., using atomic force microscopy or surface force apparatus), in situ characterization of film formation kinetics (e.g., using environmental scanning electron microscopy or diffusion wave spectroscopy), and microscopic visualization of filler–polymer interactions (e.g., using transmission electron microscopy or fluorescence resonance energy transfer). These aspects represent promising directions for future research that would further elucidate the structure–property relationships governing the performance of waterborne acrylic road marking paints.

## Figures and Tables

**Table 1:** Technical characteristics of the waterborne acrylic resin emulsion.

Appearance	pH	Density/(g·mL^−1^)	MFFT/°C	Solids Content/%
Milky white liquid	9.5	1.05	25	48~50

**Table 4:** Main technical specifications of the glass beads.

Items	Technical Requirements		Results
Appearance quality	When observed under a microscope or an optical projector, the glass beads shall be colorless spheres with smooth and perfectly round surfaces, and no visible bubbles or impurities should be present inside them.		Meet the requirements
Particle size distribution/μm	Distribution	Required mass percentage	-
	600~850	15~30	25
	300~600	30~75	62
	106~300	10~40	29
	<106	0~5	2
Sphericity/%	≥80		Meet the requirements
Density/(g·cm^−3^)	2.4~4.3 (measured: 2.52)		Meet the requirements
Refractive index	1.70~1.90		1.78
Magnetic particle content/%	≤0.1%		Meet the requirements
Water resistance	No fogging shall occur after heating in boiling water, and the amount of 0.01 mol/L hydrochloric acid required for neutralization of the glass beads shall be less than 10 mL.		Meet the requirements

**Table 5:** Factors and levels of the orthogonal experiment.

Level	Factor A	Factor B	Factor C	Factor D
1	2.0:1	10%	35%	1% (≈4% on binder solids)
2	2.4:1	15%	40%	2% (≈8% on binder solids)
3	2.8:1	20%	45%	3% (≈12% on binder solids)
4	3.2:1	25%	50%	4% (≈12% on binder solids)

**Table 6:** Performance test results of the formulations from the orthogonal experiment.

Group	A	B	C	D	Abrasion Value/mg	Hiding Power/%	Luminance Factor	Stain Resistance/%
1	1	1	1	1	5.60	88.13	0.82	2.36
2	1	2	2	2	2.88	90.96	0.83	2.02
3	1	3	3	3	3.16	91.31	0.85	2.44
4	1	4	4	4	3.84	96.00	0.85	5.41
5	2	1	2	3	4.80	81.11	0.81	2.35
6	2	2	1	4	5.80	94.32	0.83	3.60
7	2	3	4	1	5.00	93.82	0.86	1.82
8	2	4	3	2	5.40	95.57	0.83	0.50
9	3	1	3	4	4.10	86.58	0.82	1.53
10	3	2	4	3	5.18	96.60	0.83	0.91
11	3	3	1	2	3.79	98.69	0.85	0.87
12	3	4	2	1	6.24	91.48	0.86	1.70
13	4	1	4	2	4.17	94.40	0.82	0.44
14	4	2	3	1	2.77	98.24	0.81	1.74
15	4	3	2	4	3.26	96.34	0.84	1.64
16	4	4	1	3	3.33	99.59	0.85	1.78

**Table 7:** Range analysis of the orthogonal experimental results.

Indicator	Abrasion Value/mg				Hiding Power/%				Luminance Factor				Stain Resistance/%			
Factor	A	B	C	D	A	B	C	D	A	B	C	D	A	B	C	D
*k*_1_	3.87	4.67	4.63	4.90	91.60	87.55	95.18	92.92	0.838	0.816	0.835	0.838	3.06	1.67	2.15	1.91
*k*_2_	5.25	4.16	4.29	4.06	91.20	95.03	89.97	94.91	0.832	0.827	0.834	0.833	2.07	2.07	1.93	0.96
*k*_3_	4.83	3.80	3.86	4.12	93.34	95.04	92.92	92.15	0.838	0.849	0.830	0.834	1.25	1.69	1.55	1.87
*k*_4_	3.38	4.70	4.55	4.25	97.14	95.66	95.21	93.31	0.831	0.847	0.840	0.834	1.40	2.35	2.14	3.04
*R*	1.87	0.90	0.77	0.84	5.94	8.11	5.23	2.75	0.007	0.033	0.011	0.004	1.81	0.68	0.60	2.09

**Table 8:** Range analysis results of the comprehensive score vector.

Level Mean	Factor A	Factor B	Factor C	Factor D
*k*_1_	0.598	0.384	0.583	0.548
*k*_2_	0.455	0.582	0.544	0.653
*k*_3_	0.577	0.744	0.609	0.589
*k*_4_	0.724	0.645	0.618	0.564

**Table 9:** Comprehensive performance test results of the optimal formulation obtained through orthogonal experiment optimization.

Items	Test Method (Standard)	Specification Requirements	Test Results
Viscosity/KU	GB/T 9269-2009 [55]	≥70	75
Density/(g·cm^−3^)	GB/T 6750-2007 [56]	≥1.4	1.8
Non-tack drying time/min	GB/T 1728-2020 [57]	≤15	4.5
Hiding power/%	GB/T 23981.1-2019 [44]	≥90	98
Luminance factor	JT/T 280-2022 [43]	≥0.75	0.86
Abrasion value/mg	GB/T 1768-2006 [42]	≤40	2.1
Freeze–thaw stability/7 d	JT/T 280-2022 [43]	No agglomeration or skinning after freeze–thaw cycles; easy to mix uniformly.	No agglomeration or skinning after freeze–thaw cycles; easy to mix uniformly.
Water resistance	JT/T 280-2022 [43]	No abnormality after 24 h water immersion.	No blistering or peeling after 72 h water immersion.
Alkali resistance	JT/T 280-2022 [43]	No abnormality after 24 h immersion in saturated calcium hydroxide solution.	No blistering or peeling after 72 h alkali immersion.
Solid content/%	GB/T 1725-2007 [58]	≥70	82.2

## Data Availability

The data presented in this study are available on request from the corresponding author. The data are not publicly available due to privacy concerns.
